# Amaranth, the ancient pseudocereal: a promising crop for climate-resilient agriculture and healthy diets

**DOI:** 10.3389/fpls.2026.1716624

**Published:** 2026-03-13

**Authors:** Sandra M. Macías-Naranjo, José M. Arjona, Laura Huebra-Montero, Jorge Rubio-Heras, Inmaculada Sánchez-Vicente, Carlos Guillermo García-Molina, Nieves Aparicio, Pablo Albertos

**Affiliations:** 1 Innovaciones Agroalimentarias S.L., Valladolid, Spain; 2 Agricultural Research Area, Agricultural Technological Institute of Castilla and Leon (ITACyL), Valladolid, Spain; 3 Department of Botany and Plant Physiology, Excellence Research Unit of Agricultural Production and Environment, AGRIENVIRONMENT, Institute for Agribiotechnology Research, CIALE, Faculty of Biology, University of Salamanca, USAL, Salamanca, Spain; 4 TADRUS Research Group, Department of Agricultural and Forestry Engineering, University of Valladolid, Palencia, Spain

**Keywords:** abiotic stress, *Amaranthus* spp., biotic stress, climate resilience, domestication, emerging crops, genetic transformation, nutritional quality

## Abstract

*Amaranthus* spp. are plant species native to America. They are widely cultivated in tropical and subtropical regions worldwide. Some species in this genus are considered dual-crops. Their seeds and leaves can be used for both human and animal consumption. Grain and leafy amaranth are gluten-free and rich in protein, unsaturated fatty acids, vitamins, minerals, and low glycemic index carbohydrates. Traditionally, it was grown as a pseudocereal grain, especially in Central and North America. However, cultivated amaranth species still show semidomesticated traits. These traits need to be improved with current agribiotechnological methods. In this review, the actual knowledge on this emerging crop is presented, including centuries of traditional breeding techniques. The journey from history to domestication and taxonomic characterization is summarized. Furthermore, aspects of the responses to abiotic and biotic stresses of this alternative and emerging crop are analyzed in the context of climate change. Finally, the application of new genetic transformation techniques and plant breeding strategies is discussed. This provides a global perspective on the future potential of this emerging crop. However, despite all the advances made with amaranth, future challenges remain in several areas: in scientific research, which requires the full applicability of agribiotechnological methods and knowledge of the molecular basis of pest resistance and stress tolerance; in agriculture, as the optimization of agronomic practices and post-harvest management; and in the market and industry, such as marketing techniques and policies.

## 1 Introduction

Amaranth (*Amaranthus* spp.) is an ancient crop that commands attention, boasting high nutritional value and resilience across diverse agroclimatic zones. It can help strengthen food security (Malik et al., 2023). Recent studies reveal its remarkable ability to withstand abiotic stress and efficiently capture carbon through its C4 photosynthetic mechanism (Sarker and Oba, 2018b). Excelling in low-input agricultural systems, amaranth emerges as a beacon of hope for the climate challenges ahead (Malik et al., 2023; Mukuwapasi et al., 2024; Yeshitila et al., 2024; Yadav and Yadav, 2024). Despite these advantages, global amaranth production and marketing remain limited and fragmented. Market size estimates for amaranth-derived products vary: one source reports a 2024 value of USD 8.13 billion, with a projected annual growth rate of 11.7% through 2034 (Polaris Market Research, 2024), while another estimates a 2024 value of USD 12.58 billion (Maximize Market Research, 2024). These differences reflect inconsistencies in country-level production and reporting. Market expansion is driven by rising demand for functional foods, gluten-free products, and plant-based nutritional alternatives.

Regarding yield, agronomic reports indicate values ranging from 1,500 to 7,200 kg/ha under optimal conditions. This reflects its adaptability and productive potential in different regions (Mukuwapasi et al., 2024). Although the global cultivated area remains poorly documented, amaranth cultivation has gained importance in India, Nepal, China, Mexico, and East African countries. Its use as a leafy vegetable and grain has increased in recent years. In the latter region, policies promoting traditional African crops and community nutrition programs have fostered their adoption in local food systems (FAO-OCOP reports; country-specific institutional references). Building on these regional trends, in terms of recent policies, in 2025, amaranth was selected by Mexico as part of the FAO’s One Country, One Priority Product (OCOP) initiative, signaling an institutional effort to promote its sustainable production, improve its integration into value chains, and strengthen its role in national food security (Mexico Advances Sustainable Amaranth Production, 2025). This initiative reflects the renewed global interest in nutritionally dense, environmentally resilient crops.

From a nutritional perspective, amaranth is widely recognized for its high protein content and balanced essential amino acid profile. These traits distinguish it from other traditional grains and drive its increasing use in agri-food and nutraceutical products (Coelho et al., 2018; Pisarikova et al., 2005; Stevanović et al., 2024). While its cultivation is rooted in Mesoamerica and the Andes, where it played a central role in pre-Hispanic diets, its contemporary revaluation signals global interest in alternative, sustainable crops that diversify the agricultural base and mitigate risks associated with reliance on major crops (Mayes et al., 2012; Bazile et al., 2013; Das, 2016; Ruth et al., 2021).

Amaranth is a crop with deep roots and great promise. This review weaves together the latest discoveries about its rich history, impressive genetic diversity, and remarkable nutritional and stress-resilient qualities. We also explore amaranth’s rise in industry, its ongoing genetic improvement, and its rapidly evolving role in food security, sustainable agriculture, and biotechnology. Through this synthesis, we uncover gaps and new opportunities to unlock amaranth’s full global potential.

## 2 History, domestication, and characterization of crop amaranth

### 2.1 Taxonomy and diversity

**Figure 1:**
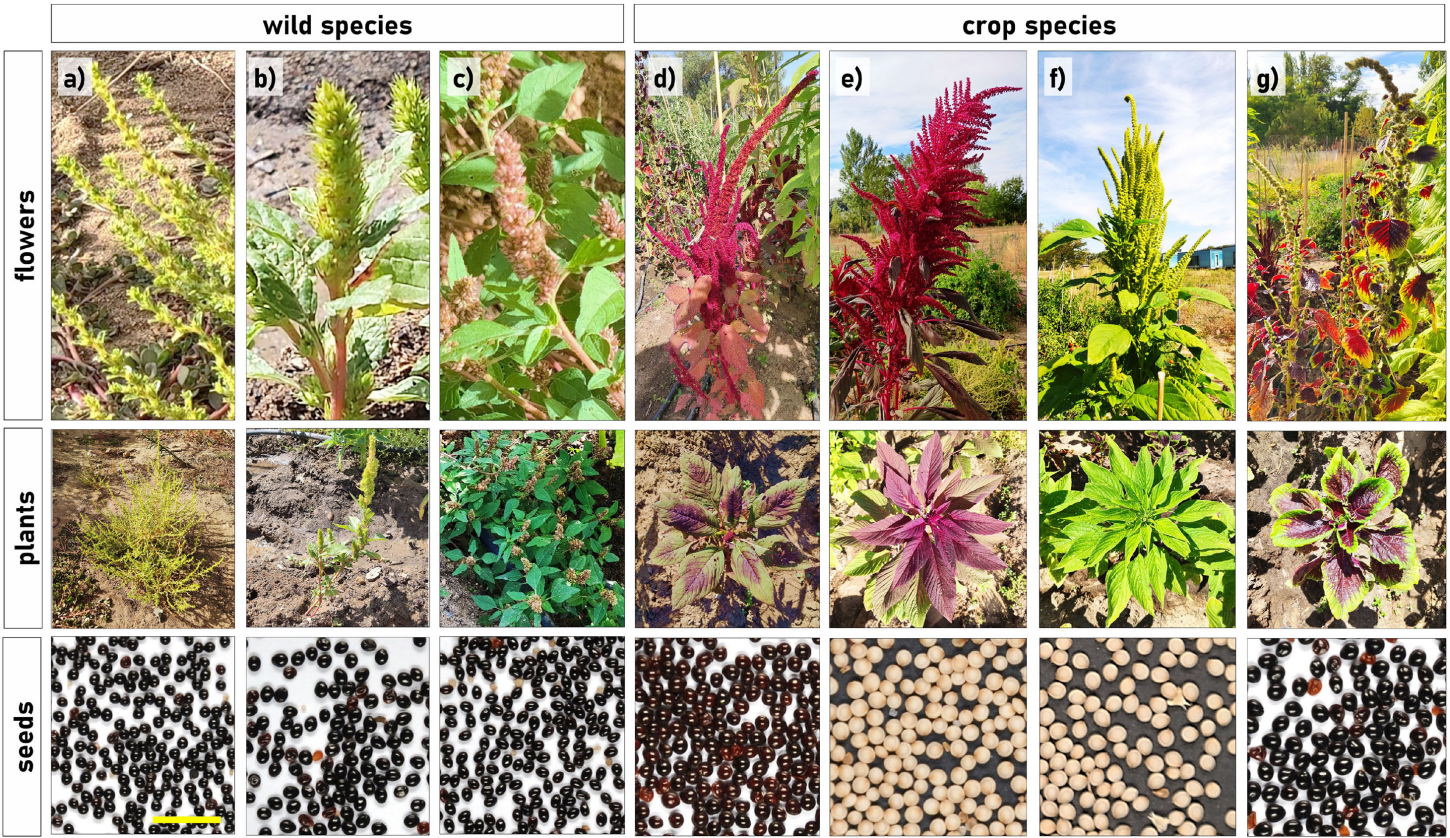
Morphological comparison with detailed inflorescence, adult plant and seed of wild and cultivated *Amaranthus species*. **(a)***A. albus*, **(b)***A. retroflexus* and **(c)***A*. *viridis* represent wild species, while **(d)***A*. *caudatus*, **(e)***A*. *hypochondriacus*, **(f)***A*. *cruentus*, and **(g)***A. tricolor* correspond to cultivated semi-domesticated species.

The genus *Amaranthus*, belonging to the family Amaranthaceae, was first described by Carl Linnaeus in 1753 and later divided into three currently accepted subgenera: *Acnida*, *Albersia*, and *Amaranthus* (Han et al., 2025; Kunth, 1838; Linné and Linné, 1753; Robertson, 1981). It is a monophyletic genus comprising approximately 75 recognized species, with 40 to 60 of them being native to the Americas, representing close to 80% of its diversity (Costea and DeMason, 2001; Sauer, 1967; Xu et al., 2022). While many *Amaranthus* species are considered weeds, as the wild species, several have been selected for crop domestication and human consumption, known as the grain amaranth or crop species (**Figure 1**) (Park et al., 2020; Ruth et al., 2021). Wild and grain species differ in plant, inflorescence and seed size. The subgenus *Amaranthus*, which includes the principal grain-producing species, is primarily native to tropical and warm-temperate regions of the Americas. It includes approximately 20 monoecious, mainly self-pollinating species, exhibiting high diversity with additional distribution across Africa, Australia, and Eurasia (Sauer, 1967).

### 2.2 Domestication and historical cultivation

Genetic studies have identified *Amaranthus hybridus* L. as the closest wild ancestor of cultivated amaranth species (Clouse et al., 2016; Stetter and Schmid, 2020). Based on this phylogenetic relationship, three major hypotheses have been proposed regarding its domestication: the monophyletic hypothesis, which suggests a single origin derived from *A. hybridus* (Sauer, 1976); the polyphyletic hypothesis, which proposes independent domestication events (Sauer, 1967); and a mixed hypothesis that considers multiple domestication events from different *A. hybridus* populations (Mallory et al., 2008). These processes, together with the species’ predominantly allogamous reproductive system, help explain the remarkable genetic heterogeneity of the genus (Stetter and Schmid, 2020), which has facilitated its diversification into distinct uses, including grain production, edible leaves, and forage (Bekkering and Tian, 2019).

Molecular and biochemical analyses have further enriched this evolutionary understanding. Techniques such as phenolic compound chromatography and Random Amplified Polymorphic DNA (RAPD), Amplified Fragment Length Polymorphism (AFLP), and Inter Simple Sequence Repeat (ISSR) markers confirm the close relationship between *A. hypochondriacus* and *A. caudatus*, and reinforce the role of *A. hybridus* as the common ancestor of the grain amaranths (Das, 2014). Currently, domesticated species are grouped into four categories based on their primary use: grain amaranths, leafy vegetables, ornamentals, and weeds. This last group includes species such as *A. retroflexus*, *A. albus*, *A. viridis*, *A. palmeri*, *A. hybridus*, *A. powellii*, and *A.* sp*inosus*, known for their strong invasive capacity (Anuradha et al., 2023), although in some regions they are also used as forage or for human consumption (Das, 2016).

Historically, amaranth was a fundamental crop for pre-Columbian civilizations, particularly the Aztec, Maya, and Inca (Bekkering and Tian, 2019). In Mexico, its use dates back 6000–8000 years, both as a staple food and in religious ceremonies (Ruth et al., 2021). During the Spanish colonization, its cultivation was banned, leading to a dramatic decline; nevertheless, it persisted within indigenous communities (Ruth et al., 2021; Flores and Teutonico, 1986). After its introduction to Europe in the 16 th century, and later to Africa and Asia, the crop remained marginal for several centuries. Scientific interest resurged in the 1970s, driven by the recognition of its high protein quality (Assad et al., 2017; Ruth et al., 2021).

Compared with other pseudocereals such as quinoa (*Chenopodium quinoa*), amaranth displays a distinct evolutionary and agronomic trajectory. A synthesis of their geographic origins, antiquity of domestication, and proposed ancestry is presented in **Table 1**. Although both crops were essential in pre-Columbian diets and declined after the conquest (Anuradha et al., 2023), their domestication histories differ. Amaranth was domesticated independently in Mesoamerica and the Andes from diverse *A. hybridus* populations (Mallory et al., 2008). Quinoa, in contrast, originated in the Andean Altiplano roughly 7000 years ago (Bhargava and Srivastava, 2013) and is derived from a tetraploid species related to *C. hircinum* (Jarvis et al., 2017). Buckwheat (*Fagopyrum* spp.), meanwhile, originates from Central Asia—with recent research placing its origin in southwestern China and the Himalayan region—and spread into Europe around 4000 BCE (Gahlaut and Jaiswal, 2024; Joshi et al., 2020).

**Table 1:** Origin, domestication and key traits of major pseudocereals.

Pseudocereal	Family	Main origin/Center of diversity	Antiquity of domestication	Progenitors and key domestication notes	Reference
Amaranth (*Amaranthus* spp.)	Amaranthaceae	Native to the American continent, with the principal grain species in Mexico and Central America (*A. cruentus*, *A. hypochondriacus*) and in the Andean region of South America (*A. caudatus*).	An ancient crop. Seeds and plants of *A. cruentus* found in Mexico (Tehuacán Valley) date to 4000 BCE. Cultivated by the Aztecs 6,000–8,000 years ago in central Mexico.	The ancestor is widely accepted as *A. hybridus*. Evidence suggests that domestication involved multiple independent events from geographically distinct subpopulations. Domestication remains incomplete, as amaranth retains small seeds prone to shattering and strong photoperiod sensitivity, despite its long cultivation history.	Das, 2014; Gonçalves-Dias et al., 2023; Joshi et al., 2020; Mallory et al., 2008; Sánchez-del Pino et al., 2025; Stetter et al., 2015; Stetter and Schmid, 2017.
Quinoa (*Chenopodium quinoa* Willd.)	Genus Chenopodium	Andean region of South America (Bolivia, Peru, Chile). The center of diversity lies in the Altiplano between Peru and Bolivia, around Lake Titicaca.	Cultivated for more than 5,000 years, with the earliest evidence of domestication ~7,000 years ago around Lake Titicaca.	Thought to have been domesticated from tetraploid species related to *C. hircinum*. *C. berlandieri* from North America is considered the basal species of the complex.	Anuradha et al., 2023; Bhargava and Srivastava, 2020; Gahlaut and Jaiswal, 2024; Jarvis et al., 2017.
Chia (*Salvia hispanica* L.)	Lamiaceae	Guatemala and southern Mexico.	Recognized as a grain in ancient civilizations. Specific archaeological dates are not extensively detailed in the literature, but it is a native Mexican seed.	Used as a pseudocereal because of its lipid- and protein-rich seeds. The genetic resource potential is more limited compared with amaranth or quinoa.	Bekkering and Tian, 2019
Buckwheat (*Fagopyrum* sp.)	Polygonaceae (Dicot).	Central Asia. Recent evidence points to the southwestern region of China and the Himalayas.	First appearance in the Balkan region of Europe around 4000 BCE.	The two cultivated species, *F. esculentum* (common buckwheat) and *F. tataricum* (tartary buckwheat), are considered domesticated in China. It is one of the three starchy grain crops that are not grasses.	Gahlaut and Jaiswal, 2024; Joshi et al., 2020.

Their contemporary trajectories are equally distinct. Quinoa has achieved widespread global adoption, bolstered by international recognition, including the 2013 International Year of Quinoa (Jarvis et al., 2017). In contrast, amaranth remains an orphan crop, largely confined to traditional growing regions, although its popularity is increasing due to its nutraceutical value (Anuradha et al., 2023).

This historical and genetic context is not free of controversy, reflecting the evolutionary complexity of the genus *Amaranthus* and its implications for breeding programs. Taxonomic classification is particularly challenging due to the pronounced morphological similarity, phenotypic plasticity, and frequent hybridization among wild and cultivated species (Das, 2014; Sánchez-del Pino et al., 2025; Stetter and Schmid, 2017).

One of the main points of debate concerns the origin of the three grain species (*A. cruentus*, *A. hypochondriacus*, *A. caudatus*) and their relationship to the wild species comprising the “*Hybridus* Complex” (Sánchez-del Pino et al., 2025). Although *A. hybridus is* widely recognized as the most likely ancestor, its polyphyletic nature complicates this interpretation (Sánchez-del Pino et al., 2025; Mallory et al., 2008). The taxonomic status of *A. quitensis* further adds complexity: some authors consider it part of *A. hybridus*, others classify it as a subspecies, and still others view it as an intermediate population involved in the domestication of *A. caudatus* (Sánchez-del Pino et al., 2025; Stetter et al., 2015); Del Pino).

Recent genomic evidence suggests a triple, independent domestication originating from geographically differentiated *A. hybridus* subpopulations (Das, 2014; Gonçalves-Dias et al., 2023; Mallory et al., 2008; Sánchez-del Pino et al., 2025). A direct consequence of this evolutionary complexity is the incomplete domestication syndrome observed in amaranth (Stetter et al., 2015). The persistence of wild-like traits, such as small seeds with spontaneous shattering and strong photoperiod sensitivity (Stetter et al., 2015), is primarily attributed to continuous gene flow between wild and cultivated populations (Gonçalves-Dias et al., 2023), which hinders the fixation of desirable traits. The genetic diversity of amaranth represents a valuable resource, although its use remains limited. At least 61 germplasm collections exist worldwide (Bekkering and Tian, 2019; Gahlaut and Jaiswal, 2024), including more than 3300 accessions in the USDA collection (Anuradha et al., 2023). Remarkably, *A. caudatus*, despite being domesticated, exhibits higher genetic diversity than several of its wild relatives, due to continuous introgression (Stetter et al., 2015). *A. hybridus* displays the highest genetic diversity within the genus, confirming its role as a primary reservoir of genetic variation (Das, 2014; Mallory et al., 2008).

Despite this potential, genetic gain in breeding has been slow. Its effective utilization depends on modern tools such as genetic mapping, DNA barcoding, high-throughput sequencing, and marker-assisted breeding. SSR and SNP markers enable the assessment of phylogenetic relationships, the selection of suitable parents, and the design of breeding strategies. Gene editing also emerges as a promising, though still incipient, tool in amaranth (Winkler et al., 2024; Anuradha et al., 2023; Bekkering and Tian, 2019; Tahimic et al., 2013). Strategies currently employed include interspecific hybridization—which increases polymorphism useful for mapping and selection—and the development of varieties oriented toward nutraceutical traits or stress tolerance (Anuradha et al., 2023). However, relevant genetic incompatibilities have been documented, such as the lethal F1 hybrids between *A. cruentus* and *A. caudatus* (Gonçalves-Dias et al., 2023), which restrict crossing possibilities and highlight the compatibility between *A. hypochondriacus and A. caudatus* as an especially promising combination.

### 2.3 Morphological and ecological characteristics

**Figure 2:**
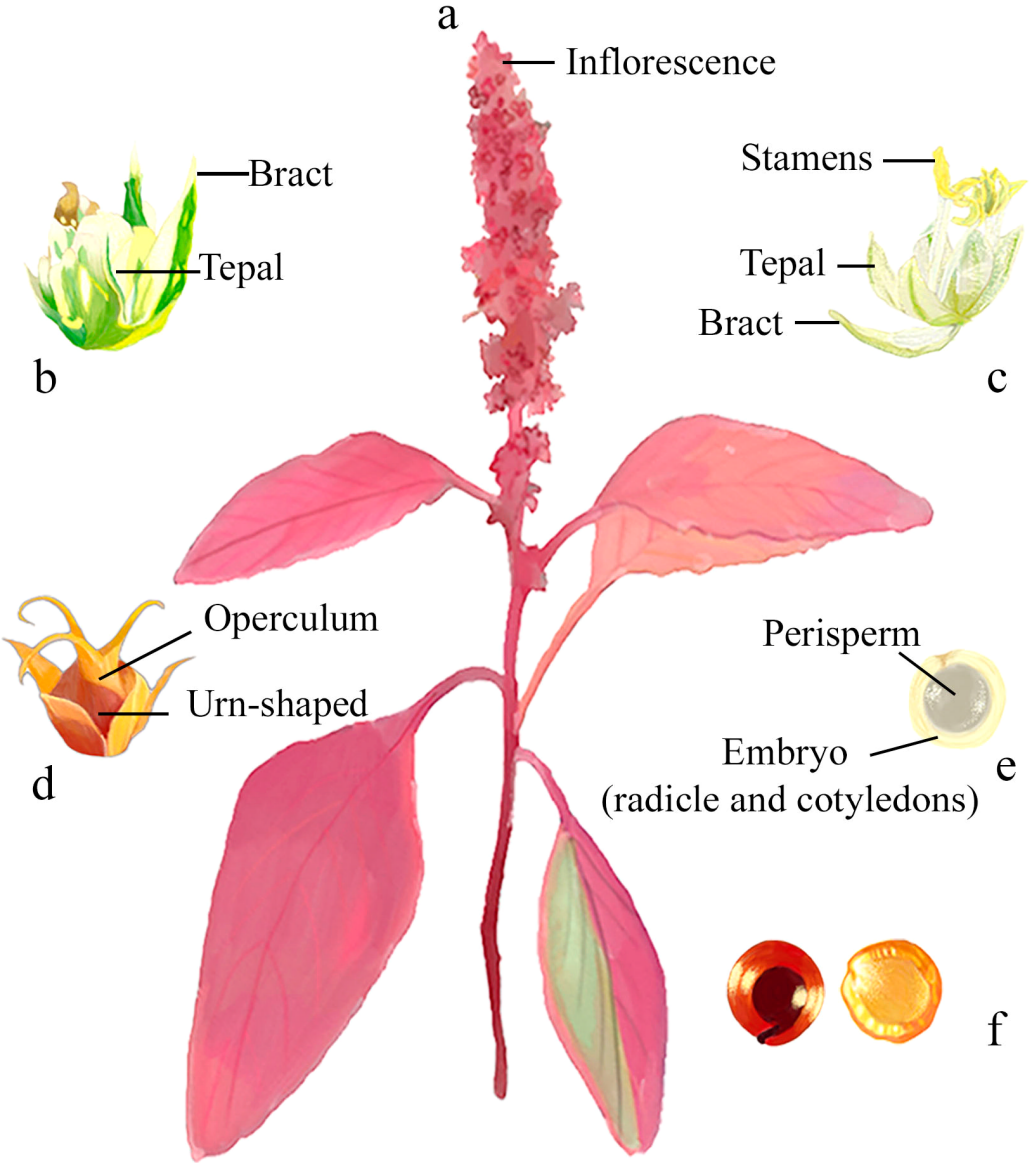
General morphology of *Amaranthus* spp. **(a)** Mature plant, **(b)** staminate flower, **(c)** pistillate flower, **(d)** fruit, **(e)** translucent immature seed, and **(f)** seed coat color variability in mature seeds.

From an ecological perspective, *Amaranthus* spp. are primarily annual herbs adapted to moist soils and capable of withstanding adverse environmental conditions. Their C4 Kranz photosynthetic metabolism confers high efficiency in light capture and water use (Assad et al., 2017; Sage et al., 2007). The genus exhibits great morphological diversity: stems vary widely in size, shape, and color—from a few centimeters to over 2 meters tall, and from pale yellow-white to vivid red or purple. Leaves are simple, alternate, and variable in shape (ovate, rhombic, linear, etc.) (Costea and DeMason, 2001). The inflorescences are complex, axillary or terminal, arranged in spikes or panicles, which may be erect or pendulous. These are composed of glomeruli formed by dichasia or cymes. The small unisexual flowers are green or reddish. Pistillate flowers typically contain 3–5 sepals, a pistil with 2–3 stigmas, and a single ovule, while staminate flowers include 3–5 stamens with dorsifixed anthers and 1–2 bracteoles, which may be spiny, foliaceous, or membranous. Male flowers are usually concentrated at the apex of the inflorescence and are fewer in number than female ones (Costea and DeMason, 2001; Costea and Tardif, 2003; Iamonico, 2023). The fruits can be indehiscent (utricles) or dehiscent (pyxidia), come in various shapes, and have a membranous pericarp; the type of dehiscence is key to distinguishing species. The seeds are lenticular or subglobose, with an annular embryo and floury perisperm, and they present a variety of colors, such as white, cream, brown, black, reddish, or pink. *Amaranthus*-type pollen is small, pantoporate, and spinulose (Bayón and Bayón, 2015). The standard chromosome number is 2n = 32 or 34 (n = 16, 17), and *A. dubius* is the only known tetraploid species (Das, 2016; Han et al., 2025). Representative morphological features of *Amaranthus*, including, mature plant, inflorescence, staminate and pistillate flowers, fruit, and seed variability are illustrated in **Figure 2**.

**Figure 3:**
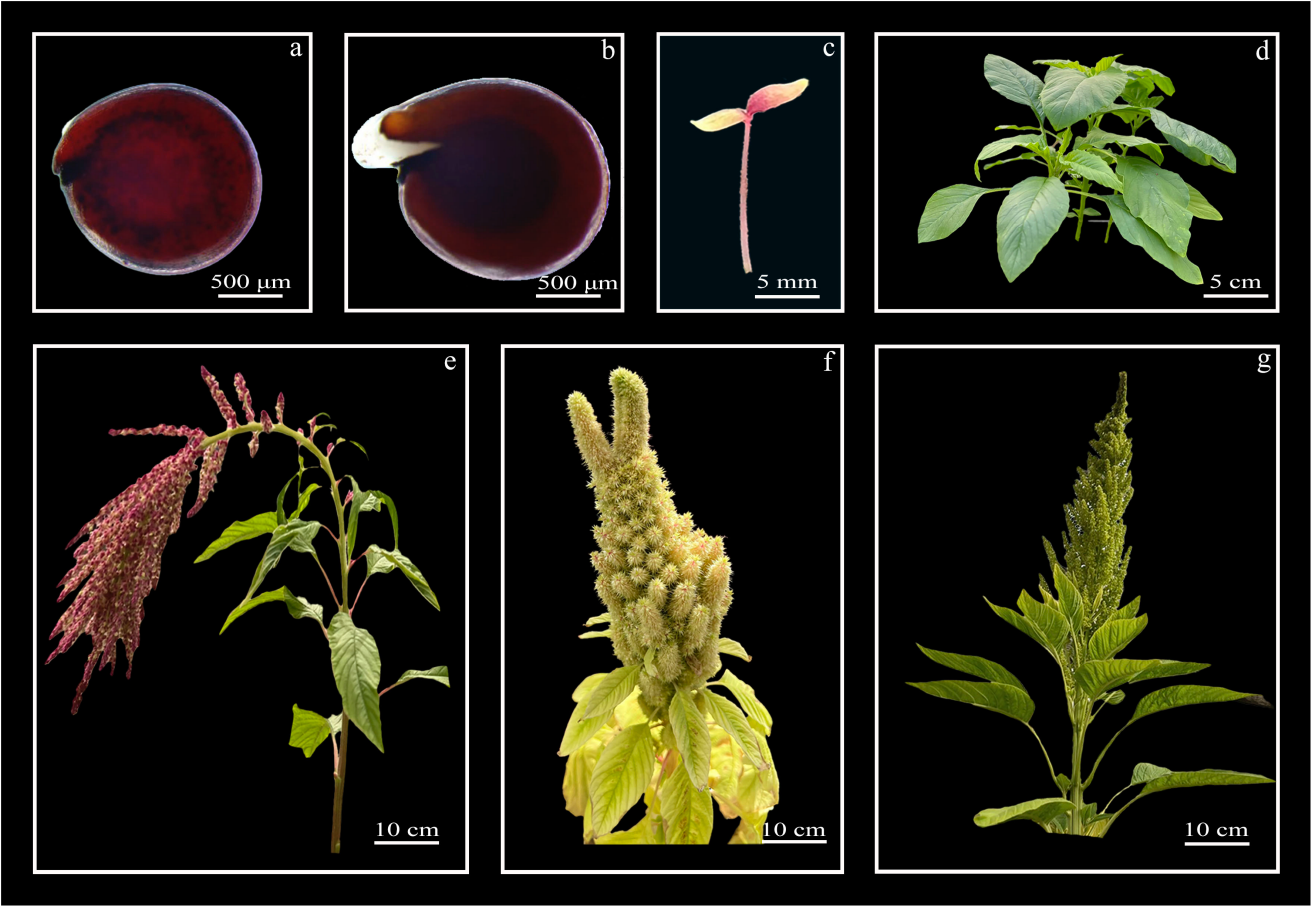
Life cycle of cultivates *Amaranthus* spp. **(a)** Seed, **(b)** germination onset (≈1 day after sowing), **(c)** seedling with fully expanded cotyledons (3 days after sowing), **(d)** seedlings at 15 days after sowing, **(e-g)** inflorescences of adult plants (60–90 days after sowing).

In addition to their morphological diversity, species of this genus have a relatively short annual life cycle, generally ranging from 90 to 150 days, depending on the species and environmental factors such as photoperiod, temperature, and nutrient availability. Plant development follows a well-defined phenological sequence, marked by germination, cotyledon expansion, vegetative growth, flowering, seed formation, and senescence (Das, 2016). This progression has been documented using standardized systems, such as the BBCH (Biologische Bundesanstalt Bundessortenamt and Chemische Industrie) code, particularly in species like *A. cruentus*, *A. hybridus*, and *A. hypochondriacus* (Martínez-Núñez et al., 2019). Some of these differential phenotypical traits are shown in **Figure 3** with photographs covering the seed-to-seed phases in cultivated *Amaranthus* such as *A. cruentus*.

## 3 Nutritional profile

Amaranth species have an outstanding nutritional profile, featuring high levels of protein, essential amino acids, minerals, vitamins, bioactive compounds, and functional lipids. These nutrients are present in both seeds and leaves, though their distribution varies between the tissues (Narwade and Pinto, 2018; Sarker et al., 2020b). The seeds are rich in high-quality protein (Akin Idowu et al., 2013), lipids containing squalene, and a starch with unique technological properties, while the leaves are especially high in micronutrients, carotenoids, vitamin C, dietary fiber, and antioxidants (Matías et al., 2024). As a result, amaranth is notable not only for its direct nutritional benefits but also for its potential as a functional food and a resource to help address global food security and malnutrition issues (Matías et al., 2024; Narwade and Pinto, 2018; Sarker and Oba, 2018a; Sarker et al., 2020a; Thakur et al., 2021). **Tables 2A** and **2B** show the composition of the different nutritional components of amaranth seeds and leaves, respectively.

**Table 2A:** Nutritional composition, bioactive compounds, and antinutritional factors in seeds of *Amaranthus* species.

Profile	Component	Species/variety	Concentration range	Unit	Reference
Nutritional composition	Protein	*A. caudatus*	13.1–15.4	g/100 g DW	B
	Protein	*A. caudatus*	11.50–19.80	% (DW)	A
	Fat/oil	*A. caudatus*	8.08–9.50	g/100 g DW	B
	Oil content	*A. cruentus*	7.01	% (w/w)	D
	Carbohydrates	*A. caudatus*	69.8–72.6	g/100 g DW	B
	Crude fiber	*A. caudatus*	3.60–4.59	g/100 g DW	B
	Dietary fiber	*A. molleros, A. caudatus, A. mantegazzianus*, and *A. cruentus*	8.4	g/100 g	C
	Moisture	*A. molleros, A. caudatus, A. mantegazzianus*, and *A. cruentus*	8.5	g/100 g	C
Amino acids	Essential amino acids (sum)	*A. cruentus*	29.67	g/100 g protein	D
	Lysine (Lys)	*A. cruentus*	3.2–14.32	g/100 g protein	D
	Methionine (Met)	*A. cruentus*	1.77–7.5	g/100 g protein	D
	Cysteine (Cys)	*A. cruentus*	0.98–6.06	g/100 g protein	D
	Leucine (Leu)	*A. cruentus*	5.33–12.25	g/100 g protein	D
	Valine (Val)	*A. cruentus*	3.11–9.90	g/100 g protein	D
	Isoleucine (Ile)	*A. cruentus*	2.87–4.2	g/100 g protein	D
	Histidine (His)	*A. cruentus*	2.2–6.83	g/100 g protein	D
	Arginine (Arg)	*A. cruentus*	6.70–12.15	g/100 g protein	D
	Phenylalanine (Phe)	*A. cruentus*	3.80–8.36	g/100 g protein	D
	Threonine (Thr)	*A. cruentus*	2.5–5.76	g/100 g protein	A
Mineral profile	P	*A. caudatus, A. molleros, A. cruentus*, *A. mantegazzianus*	339–577	mg/100 g DW	B, C
	Na	*A. caudatus*	1.82–3.50	mg/100 g DW	B
	K	*A. caudatus, A. molleros, A. cruentus*, *A. mantegazzianus*	482–531	mg/100 g DW	B, C
	Ca	*A. caudatus, A. molleros, A. cruentus*, *A. mantegazzianus*	63.9–285	mg/100 g DW	B, C
	Mg	*A. caudatus, A. molleros, A. cruentus*, *A. mantegazzianus*	206–336	mg/100 g DW	B, C
	Zn	*A. caudatus, A. molleros, A. cruentus*, *A. mantegazzianus*	2.9–4.67	mg/100 g DW	B, C
	Mn	*A. caudatus*	1.88–5.90	mg/100 g DW	B
	Fe	*A. caudatus*	7.52–14.8	mg/100 g DW	B
	Cu	*A. caudatus*	0.74–1.23	mg/100 g DW	B
	Se	*A. molleros, A. caudatus, A. mantegazzianus*, and *A. cruentus*	18.7	mg/100 g (Se in µg/100 g)	B, C
Fatty acid profile	Linoleic	*A. cruentus*	44.2–48.6	% of total FA	F
	Oleic	*A. cruentus*	23.4–28.6	% of total FA	F
	Stearic	*A. cruentus*	4.0–4.2	% of total FA	F
	Palmitic	*A. cruentus*	19.2–20.2	% of total FA	F
	Linolenic	*A. cruentus*	0.6–1.1	% of total FA	F
Phenolic acids profile	Vanillic	*A. cruentus*	10.33	mg/kg seed	D
	Caffeic	*A. cruentus*	3.37	mg/kg seed	D
	Chlorogenic	*A. cruentus*	4.00	mg/kg seed	D
	*p*-Coumaric	*A. cruentus*	3.63	mg/kg seed	D
	Rosmarinic	*A. cruentus*	7.68	mg/kg seed	D
	Myricetin	*A. cruentus*	105.05	mg/kg seed	D
	Luteolin	*A. cruentus*	39.15	mg/kg seed	D
	Kaempferol	*A. cruentus*	1041.50	mg/kg seed	D
	Total	*A. cruentus*	1214.71	mg/kg seed	D
Vitamin profile	Vitamin C	*A. molleros, A. caudatus, A. mantegazzianus*, and *A. cruentus*	4.2	mg/100 g	C
	Vitamin B1 (Thiamine)	*A. molleros, A. caudatus, A. mantegazzianus*, and *A. cruentus*	0.07–0.1	mg/100 g	C
	Vitamin B2 (Riboflavin)	*A. molleros, A. caudatus, A. mantegazzianus*, and *A. cruentus*	0.19–0.23	mg/100 g	C
	Vitamin B3 (Niacin)	*A. molleros, A. caudatus, A. mantegazzianus*, and *A. cruentus*	0.9–1.45	mg/100 g	C
	Vitamin B5 (Pantothenic acid)	*A. molleros, A. caudatus, A. mantegazzianus*, and *A. cruentus*	1.5	mg/100 g	C
	Vitamin B6 (Pyridoxine)	*A. molleros, A. caudatus, A. mantegazzianus*, and *A. cruentus*	0.6	mg/100 g	C
	Vitamin B9 (Folate)	*A. molleros, A. caudatus, A. mantegazzianus*, and *A. cruentus*	82.2	µg/100 g	C
	*Choline (Vitamin B12)*	*A. molleros, A. caudatus, A. mantegazzianus*, and *A. cruentus*	69.8	mg/100 g	C
	Vitamin A (Retinol)	*A. molleros, A. caudatus, A. mantegazzianus*, and *A. cruentus*	2.1	IU/100 g	C
	Tocopherols (vitamin E; α/β/δ)	*A. molleros, A. caudatus, A. mantegazzianus*, and *A. cruentus*	α 1.19; β 0.96; δ 0.69	mg/100 g	C
Bioactive lipids (unsaponifiable fraction)	Squalene (seed; DW)	*A. hypochondriacus, A. caudatus*	1.6–4.7	mg/g DW	E
	Squalene (raw seeds)	*Amaranthus* spp.	3.2–7.7	%	A
	Squalene (oil)	*A. cruentus* (oil)	4.9–5.3	g/100 g oil	F
Antioxidant profile	β-Sitosterol	*A. cruentus* oil	766.3–786.2	mg/100 g oil	F
	Campesterol	*A. cruentus* oil	28.8–30.8	mg/100 g oil	F
	Stigmasterol	*A. cruentus* oil	11.7–48.8	mg/100 g oil	F
	Δ7-Avenasterol	*A. cruentus* oil	275.9–334.8	mg/100 g oil	F
Antinutritional profile	Phytic acid	*Amaranthus* spp.	0.61–1.34	g/100 g	G
	Saponins	*Amaranthus* spp.	5.3–10.7	mg/100 g	G
	Protease inhibitors	*Amaranthus* spp.	79–186	HU/mg	G
	Lectins	*Amaranthus* spp.	0.52–1.02	TIU/mg	G
	Oxalates	*Amaranthus* spp.	46–278	mg/100 g	G

A = Verma (2022); B = Mérida-López et al. (2023); C = Stevanović et al. (2024); D = Procopet and Oroian (2022);E= Gupta et al. (2023); F = Sayed-Ahmad et al. (2022); G = Matías et al. (2024); H = Sidorova et al. (2023); I = Sarker & Oba (2019); J = Sarker et al. (2020a); K = Sarker & Oba (2019); L= Jahan et al. (2022).

**Table 2B:** Nutritional composition, bioactive compounds, and antinutritional factors in leaves of Amaranthus species.

Profile	Component	Species/variety (grouped)	Concentration range	Unit	Reference
Nutritional composition	Protein	*A. tricolor, A. viridis, A. hypochondriacus*, *A. spinosus*	3.1–26.6	g/100 g (FW–DW)	I, J, C, L
	Fat/oil	*A. tricolor, A. viridis*, *A. spinosus*	0.2–0.8	g/100 g FW	I, C, L
	Carbohydrates	*A. tricolor, A. viridis*, *A. hypochondriacus*	2.3–9.3	g/100 g FW	I, C, L
	Crude fiber	*A. tricolor, A. viridis*, *A. spinosus*	0.54–6.83	g/100 g FW	I, L
	Dietary fiber	*A. tricolor*	6.0–10.8	g/100 g FW	J
	Moisture	*Amaranthus* spp.	81.7–86.8	g/100 g FW	J
Amino acids	Essential amino acids (sum)	*A. tricolor, A. hypochondriacus*	31–38	g/100 g protein	C, K
	Lysine (Lys)	*A. tricolor, A. hypochondriacus*	3.8–7.3	g/100 g protein	C, K
	Methionine (Met)	*A. tricolor, A. hypochondriacus*	3.2–7.5	g/100 g protein	C, K
	Cysteine (Cys)	*A. tricolor, A. hypochondriacus*	2.7–4.8	g/100 g protein	C, K
	Leucine (Leu)	*A. tricolor, A. hypochondriacus*	4.5–7.5	g/100 g protein	C, K
	Valine (Val)	*A. tricolor, A. hypochondriacus*	3.9–6.4	g/100 g protein	C, K
	Isoleucine (Ile)	*A. tricolor, A. hypochondriacus*	2.7–5.2	g/100 g protein	C, K
	Histidine	*A. tricolor, A. hypochondriacus*	2.2–3.6	g/100 g protein	C, K
	Arginine	*A. tricolor, A. hypochondriacus*	7.9–11.6	g/100 g protein	C, K
	Phenylalanine (Phe)	*A. tricolor, A. hypochondriacus*, *A. viridis*	3.1–5.1	g/100 g protein	C, K, L
	Threonine (Thr)	*A. tricolor, A. hypochondriacus*	2.5–4.1	g/100 g protein	C, K
Mineral profile	P	*A. tricolor, A. viridis*	68–94	mg/100 g FW	I, J
	Na	*A. tricolor, A. viridis*, *A. spinosus*	2.5–30.3	mg/100 g FW	I
	K	*A. tricolor, A. viridis*, *A. hypochondriacus*	225–758	mg/100 g FW	I, J, L
	Ca	*A. tricolor, A. viridis*, *A. spinosus*	205–524	mg/100 g FW	I, J
	Mg	*A. tricolor, A. viridis*	247–472	mg/100 g FW	I
	Fe	*A. tricolor, A. viridis*, *A. spinosus*	1.5–3.2	mg/100 g FW	I, J
Phenolic acids	Vanillic	*A. tricolor*	2.0–9.8	µg/g FW	K
	Caffeic	*A. tricolor*	0.15–1.56	µg/g FW	K
	Chlorogenic	*A. tricolor*	3.03–9.86	µg/g FW	K
	*p*-Coumaric	*A. tricolor*	0.07–1.16	µg/g FW	K
Flavonoids	Myricetin	*A. tricolor*	2.22–4.25	µg/g FW	K
	Kaempferol	*A. tricolor*	1.54–3.37	µg/g FW	K
	Total phenolics	*A. tricolor, A. hypochondriacus*	11.24–46.72	µg GAE/g FW	J, K
Vitamin profile	β-Carotene (provitamin A)	*A. viridis, A. spinosus*, *A. hypochondriacus*	4.83–64.22	mg/100 g FW	I, J
	Vitamin C	*A. tricolor, A. hypochondriacus*, *A. viridis*	12–185	mg/100 g FW	I, J
Antioxidant profile	TAC (DPPH)	*A. tricolor*	44.85	µg TE/g DW	J
	TAC (ABTS)	*A. tricolor*	33–82.55	µg TE/g DW	J
Antinutritional profile	Oxalates	*Amaranthus* spp. (leaves)	178–278	mg/100 g FW	G

A = Verma (2022); B = Mérida-López et al. (2023); C = Stevanović et al. (2024); D = Procopet and Oroian (2022);E= Gupta et al. (2023); F = Sayed-Ahmad et al. (2022); G = Matías et al. (2024); H = Sidorova et al. (2023); I = Sarker & Oba (2019); J = Sarker et al. (2020a); K = Sarker & Oba (2019); L= Jahan et al. (2022).

### 3.1 Amaranth seeds

Amaranth seeds have a notable nutritional profile within the pseudocereal group, featuring high protein levels, a lipid part rich in unsaturated fatty acids and squalene, carbohydrates with unique technological properties, and a diverse bioactive matrix (Bekkering and Tian, 2019; Chauhan et al., 2023; Park et al., 2020; Ruth et al., 2021; Singh and Punia, 2020).

#### 3.1.1 Proteins and amino acids

Amaranth seeds contain 13% to 22% protein, higher than most traditional cereals, such as wheat, maize, and sorghum (Singh and Punia, 2020). Similarly, amaranth’s quality stems from a complete essential amino acid profile, providing all nine essential amino acids at or above FAO reference levels for children and adults, whereas traditional cereals often lack such profiles. The high lysine content (up to 10.10 g/100 g protein) is especially notable, as wheat, maize, and sorghum are typically low in lysine (Malik et al., 2023; Procopet and Oroian, 2022; Soriano-García et al., 2018; Szabóová et al., 2020). Amaranth proteins are dominated by albumins (water-soluble proteins) and 11S globulins (storage proteins found in seeds), which represent between 44% and 62% of the total protein (Akin Idowu et al., 2013; Tandang-Silvas et al., 2012). The AmA1 albumin exhibits excellent digestibility and a balanced profile of essential amino acids, while 11S globulin (also called amarantin) constitutes the main storage protein and has high levels of lysine and sulfur amino acids, which are usually limiting in cereals and legumes (Luna-Suárez et al., 2010; Tandang-Silvas et al., 2012).

Analyses of various accessions show that essential amino acids contribute 31–45 g/100 g of protein. These accessions exhibit high levels of lysine, threonine, and methionine, with marginal limitations only in tryptophan or leucine, depending on genotype (Akin Idowu et al., 2013; Kumar et al., 2024). Metabolomic studies confirm these findings, reporting high concentrations of lysine, arginine, GABA, and tryptophan (Gupta et al., 2023).

Amaranth protein is highly digestible (~90%) and has a PDCAAS of ~0.72. This score is comparable to that of soybeans and higher than most pseudocereals (grain-like seeds from non-grass plants) and cereals (Narwade and Pinto, 2018; Sidorova et al., 2023). Because its prolamin content is low (<1%)—prolamins are storage proteins that can trigger reactions in celiac disease—amaranth protein is suitable for people with celiac disease. Its low allergenicity also makes it ideal for producing gluten-free products (Stevanović et al., 2024; Zhu, 2023).

Functionally, amaranth 11S globulin (a seed storage protein) helps emulsify mixtures because it has both water-loving (hydrophilic) and water-repelling (hydrophobic) regions. Its solubility depends on pH and is typical of legume proteins (Aguilar-Padilla et al., 2023; Hadidi et al., 2024; Tandang-Silvas et al., 2012). When enzymes break down these proteins, they release small fragments (peptides). These peptides may help reduce blood pressure or fight microbes, making them valuable for health foods (Cruz-Morán et al., 2023; Luna-Suárez et al., 2010; Nardo et al., 2020; Vecchi and Añón, 2009). Furthermore, amaranth’s three-strand structure (the protein’s quaternary structure), its hypervariable regions (parts of the protein that vary significantly between versions), and the existence of specialized isoforms (slightly different versions of the same protein) all contribute to its usefulness in protein engineering. For instance, these features have enabled the development of transgenic potato and maize lines with improved protein quality, as well as recombinant variants with ACE-inhibitory activity (the ability to block angiotensin-converting enzyme, which regulates blood pressure) up to eight times greater than that of the native form (Aguilar-Padilla et al., 2023; Luna-Suárez et al., 2010; Tandang-Silvas et al., 2012).

Amaranth’s protein content (13.0–17.6%) is comparable to quinoa (13.1–18.7%). Both are close to wheat, but typically lower than chia (15–25%) (Matías et al., 2024). Amaranth and quinoa have highly balanced amino acid profiles: amaranth contains 4.6–6.4 g lysine/100 g protein and 2.6–5.5 g sulfur-containing amino acids/100 g protein. These values generally exceed those of quinoa, soybeans, and other legumes, and clearly surpass wheat, rice, oats, and corn (Akin Idowu et al., 2013; Singh and Punia, 2020; Tandang-Silvas et al., 2012). Buckwheat also has relatively high lysine content, though it is less digestible due to antinutritional factors (Matías et al., 2024). The protein profile of amaranth has key implications for human nutrition. Its high lysine content makes it an excellent complement to cereal-based diets and a valuable ingredient in vegetarian and pediatric formulations or nutritional recovery products (Narwade and Pinto, 2018). Most studied accessions exceed FAO/WHO (1991) essential amino acid requirements for children aged 2–5 years (Akin Idowu et al., 2013), although some reports note tryptophan or valine may be limiting in certain samples (Sidorova et al., 2023). Taken together, these values confirm that amaranth ranks among the highest-quality plant-based protein sources within the grain and pseudocereal group.

The potential of amaranth proteins has also been demonstrated through genetic engineering. The introduction of the AmA1 gene into potatoes significantly increased the protein content of the tubers, while the expression of 11S globulin in maize improved the protein quality of the grain without inducing allergic reactivity (Adhikary et al., 2020; Luna-Suárez et al., 2010). More advanced modifications have enhanced their nutraceutical value: the insertion of four Val-Tyr dipeptide repeats into the third hypervariable region of the acid subunit generated a recombinant protein whose ACE inhibitory activity was eight times greater than that of the native protein (Luna-Suárez et al., 2010). These advances consolidate amaranth as a versatile protein platform, suitable both for the development of functional foods and for obtaining bioactive peptides of biomedical interest.

#### 3.1.2 Carbohydrates

Carbohydrates constitute between 60% and 70% of the grain’s dry matter, with starch as the main component (60–65%). Amaranth starch is characterized by its very small granule size (0.8–3 μm), high crystallinity, and genetic variability in amylose content, which allows for the differentiation of waxy and non-waxy genotypes (Zhu, 2023). Waxy genotypes have lower amylose content and rapid digestibility, making them useful for infant or sports nutrition formulations. Conversely, non-waxy genotypes have higher amylose content and a greater capacity to form type II resistant starch, with benefits for intestinal health, a reduction in the glycemic index, and prebiotic effects (Zhu, 2023). Technological processes such as popping increase the fraction of inaccessible or retrograded starch, increasing the resistant starch content and expanding the functional potential of the grain (Gupta et al., 2023; Montoya-Rodríguez et al., 2025; Reddy et al., 2023; Valadez-Vega et al., 2022). Waxy amaranth phenotypes have been identified in several important grain amaranth species, resulting from specific mutations in the *WX* locus (Park et al., 2010). These variants have been described in *A. cruentus*, *A. hypochondriacus* and *A. caudatus*, which carry the *WX-cr*, *WX-hy* or *WX-ca* alleles, respectively. All of them are characterized by a significantly reduced amylose content (approximately 3% to 6%). As a result, their starches exhibit the cohesive, highly gelatinized and film-forming properties typical of waxy starches, in contrast to the starches with higher amylose content found in standard genotypes. The fiber content of amaranth (10–25%) is similar to that of quinoa (9.4–22.7%). However, buckwheat (20–26%) and chia (18.0–35.5%) have higher values, reflecting differences in grain structure and functional behavior (Matías et al., 2024).

#### 3.1.3 Lipids, fatty acids and squalene

The lipid fraction of amaranth seeds has a remarkable nutritional and functional profile. Fat content ranges from 1.7% to 10.3%, with a high proportion of polyunsaturated and unsaturated fatty acids, representing between 74% and 83% of the total (Narwade and Pinto, 2018). Among the fatty acids, linoleic acid (C18:2, ω-6), oleic acid (C18:1), and, to a lesser extent, palmitic acid (C16:0) predominate, forming a profile consistent with beneficial effects on cardiometabolic health (Mérida-López et al., 2023; Procopet and Oroian, 2022).

A distinctive feature of amaranth is that it is considered the richest known plant source of squalene, an unsaponifiable triterpenoid hydrocarbon and precursor of sterols in plants and animals (Amare et al., 2021; Kraujalis et al., 2013; Kraujalis and Venskutonis, 2013; Ott et al., 2015; Sayed-Ahmad et al., 2022; Srivastava et al., 2021; Szabóová et al., 2020). In raw seeds, values ranging from 13 to 560 mg/100 g have been reported, with an approximate average of 213 mg/100 g (Castañeda-Reyes et al., 2021), while in the oil, squalene represents between 2.4% and 8% of the total weight, reaching higher values ​​in enriched fractions or in specific varieties (Kraujalis et al., 2013; Kraujalis and Venskutonis, 2013; Sayed-Ahmad et al., 2022). In Ethiopian varieties, the content can exceed 480 mg/100 g (Amare et al., 2021), while in *A. cruentus* (‘Pribina’), proportions close to 7% of the oil have been recorded. In addition to their energetic and structural roles, amaranth lipids are important sources of essential fatty acids (ω6 and ω3), necessary for the digestion, absorption, and transport of fat-soluble vitamins (A, D, E, and K).

Technological processes, such as popping, significantly increase the concentration of squalene, reaching values of 8.13% in popped grain (Reddy et al., 2023; Srivastava et al., 2021; Verma, 2022). Extraction methods such as supercritical CO_2_ extraction yield highly enriched fractions, reaching values of around 17.9 g/100 g of oil when pressure gradient separators are used (Kraujalis et al., 2013; Kraujalis and Venskutonis, 2013; Toimbayeva et al., 2025). Squalene offers profound nutraceutical benefits, including antioxidant activity, emollient properties, hypolipidemic effects, and photoprotective properties against UV radiation (Amare et al., 2021; Park et al., 2020; Sayed-Ahmad et al., 2022; Srivastava et al., 2021; Szabóová et al., 2020; Toimbayeva et al., 2025). It is also widely used as an adjuvant in vaccines, such as in the MF59 emulsion, and as a vehicle in lipophilic drug delivery systems, including liposomes and lipid nanoparticles (Castañeda-Reyes et al., 2021; Verma, 2022).

Comparatively, chia has the highest fat content among pseudocereals (25–40%), followed by amaranth, which provides 4.2–8.5% high-quality fat, and buckwheat, with a lower content (1.5–6.5%). Differences are also notable in fatty acid composition: quinoa contains 88–90% unsaturated fatty acids, of which 66–68% are polyunsaturated, with linoleic acid being the most prevalent (58.5–61.4%). Amaranth, on the other hand, has less linoleic acid (35.2–37.3%), but higher proportions of oleic acid (35.4–36.7%) and a considerably higher amount of palmitic acid (21%) compared to quinoa (9.0–10.4%). These differences are reflected in the ω6:ω3 ratio, significantly higher in amaranth (27.5–36.1:1) than in quinoa (6–9:1) and much lower than in chia (0.3–0.4:1), the latter being rich in α-linolenic acid (55.2–65.9%) (Matías et al., 2024).

Regarding squalene, the different sources show marked variations in their concentration: shark liver oil is traditionally the richest source, with a yield equivalent to 40% of the liver weight or between 2300 and 8400 mg/100 g of oil (Castañeda-Reyes et al., 2021; Sayed-Ahmad et al., 2022; Szabóová et al., 2020), olive oil, the only plant crop used commercially to extract squalene, contains around 0.4% in its oil (150–747 mg/100 g) (Sayed-Ahmad et al., 2022; Srivastava et al., 2021; Szabóová et al., 2020), rice bran oil provides 0.3% (Srivastava et al., 2021). Pseudocereals such as quinoa and buckwheat reach only 58.4 mg/100 g and 1.9 mg/100 g of dry matter, respectively (Amare et al., 2021). In this context, amaranth stands out as the most cost-effective plant-based alternative, with clear advantages over other vegetable oils, as well as over other pseudocereals.

#### 3.1.4 Minerals

The seeds also provide significant amounts of essential minerals, such as calcium (Ca), phosphorus (P), magnesium (Mg), zinc (Zn), and manganese (Mn), with considerable variation among species and accessions (Ruth et al., 2021; Singh and Punia, 2020). Amaranth is generally one of the richest sources of minerals among pseudocereals, with higher levels of P, Ca, and Mg than quinoa, although lower in potassium (K) (Matías et al., 2024). Its calcium content can be up to three times higher than that of quinoa and surpasses that of plant-based foods known for their calcium content, such as almonds or legumes (Schmidt et al., 2023; Ramírez-Ojeda et al., 2018). Buckwheat has a total mineral content of 1.7–2.7% ash, slightly lower than that of amaranth and quinoa. Chia provides 4.0–4.8% ash, with a calcium content even higher than that of milk (Ullah et al., 2016; Muñoz et al., 2013).

#### 3.1.5 Other nutritional and antinutritional molecules

Amaranth seeds contain antinutritional compounds such as phytic acid, saponins, tannins, trypsin inhibitors, and lectins. Phytic acid can range from 2.9 to 7.9 g/kg and reduces the bioavailability of minerals such as Fe, Zn, and Ca, although treatments such as soaking, germination, fermentation, and extrusion significantly decrease its content (Thakur, 2021; Kumar et al., 2024; Narwade and Pinto, 2018). Interestingly, saponins and tannins show wide variability among cultivars and, although traditionally classified as antinutrients, they also perform antioxidant and cholesterol-lowering functions at moderate concentrations, thus being considered compounds with a dual role: antinutritional and bioactive (Narwade and Pinto, 2018).

Comparatively, amaranth has moderate levels of phytic acid (2.9–7.9 g/kg), lower than those of quinoa (10.5–13.5 g/kg) and much lower than those of buckwheat (35–38 g/kg). It also contains saponins at levels of 0.9–4.9 mg/kg, clearly lower than those of quinoa (6.3–692.5 mg/kg) (Matías, 2023). Trypsin inhibitors and lectins are present in low quantities in the grain; Highly concentrated extracts may exhibit cytotoxicity *in vitro*, but levels in processed foods are safe, as heat treatments drastically reduce their activity (Valadez-Vega et al., 2021, 2022). Overall, the antinutritional compounds in amaranth are within safe ranges and can be modulated by processing, allowing their bioactive benefits to be preserved without compromising nutrient bioavailability.

### 3.2 Amaranth leaves

The leaves of *Amaranthus* spp. are vegetables of high nutritional and phytochemical value. They significantly complement the seeds’ nutritional profile. While the grain concentrates storage proteins, starch, and functional lipids, the leaves are distinguished by high micronutrient density. They also contain water-soluble and fat-soluble vitamins, as well as a wide variety of phenolic compounds and antioxidant pigments. Composition varies across species, cultivars, and environmental conditions. However, amaranth leaves are among the most nutritious green leafy vegetables within their taxonomic group (Aderibigbe et al., 2022; Ruth et al., 2021).

#### 3.2.1 Proteins, amino acids and micronutrients

The protein content of amaranth leaves is remarkably high for a leafy vegetable (Sarker et al., 2020a; Sarker and Oba, 2019a, b). In *A. tricolor*, values close to 26.6% on a dry matter basis have been reported, while in *A. caudatus* and *A.* sp*inosus*, levels range between 15–22%, significantly exceeding those of vegetables such as spinach, chard, or lettuce (Jahan et al., 2022; Stevanović et al., 2024). Its essential amino acid profile is well balanced (Matías et al., 2024; Sarker et al., 2020b; Stevanović et al., 2024). Notable levels include lysine, threonine, and methionine (Akin Idowu et al., 2013; Sarker et al., 2020a; Sarker et al., 2020b). Depending on the genotype, amaranth leaves contain 3.8-7.3 g/100 g of protein for lysine and 3.2-7.5 g/100 g for methionine (Stevanović et al., 2024). This helps supplement cereal-based diets and is especially valuable where the leaves are the main form of amaranth consumption (Akin Idowu et al., 2013; Narwade and Pinto, 2018; Sarker et al., 2020b; Sarker and Oba, 2019b).

Regarding minerals, amaranth leaves have particularly high concentrations of K, Ca, Mg, and Fe compared to many other leafy vegetables. For example, in *A. tricolor*, potassium content reaches about 1080 mg/100 g (Jahan et al., 2022). High levels of Ca and Fe, measured on a dry weight basis, are observed in *A. lividus* and *A.* sp*inosus* (Sarker et al., 2020b). Some accessions of *A. hypochondriacus* contain over 180 mg/100 g of vitamin C (fresh weight). They also have significant amounts of β-carotene (82.34 mg/100 g FW in *A. hypochondriacus*) and other carotenoids (Sarker and Oba, 2019b, 2020). Amaranth leaves of unspecified species can provide up to 20 times more Ca, 13 times more vitamin C, 7 times more Fe, and 18 times more β-carotene than lettuce on a fresh weight basis. They can also supply up to 5 times more iron than wheat on a dry weight basis (Narwade and Pinto, 2018).

#### 3.2.2 Phytochemicals, pigments and antioxidant properties

The leaves of *Amaranthus* species are especially rich in flavonoids, phenolic acids, betalains, and carotenoids, making them among the most potent leafy vegetables in terms of antioxidants. In *A. lividus*, total phenols exceed 228 μg GAE/g. In *A.* sp*inosus*, flavonoids reach about 179 μg RE/g (Sarker Daramy et al., 2020). In *A. tricolor*, approximately 24 phenolic compounds have been identified, including nine major flavonoids: quercetin, isoquercetin, hyperoside, rutin, kaempferol, myricetin, apigenin, catechin, and naringenin. Rutin alone can reach nearly 3%, which is notable for a leafy vegetable (Sarker and Oba, 2019b; Thakur, Kumar, Ahmed, et al., 2021). Red genotypes of *A. tricolor* show the highest concentrations of total flavonoids (TFC) and phenolic acids. For example, genotype VA13 has the highest TFC, followed by VA3. Major phenolic acids found include vanillic, salicylic, protocatechuic, gallic, ellagic, chlorogenic, sinapic, p-coumaric, caffeic, and ferulic acids (Sarker and Oba, 2019b).

Amaranth leaves are also an excellent source of β-carotene, a precursor of vitamin A. In *A. hypochondriacus*, β-carotene can reach 82.34 mg/100 g FW, with an average of 58.26 mg/100 g FW (Sarker and Oba, 2019b, 2020). *A. viridis* genotypes, such as WAV7, also show high values, reaching 64.22 mg/100 g FW (Sarker and Oba, 2019b). β-carotene positively and significantly correlates with total antioxidant capacity (TAC). This relationship is seen consistently in the leaves of several amaranth species (Sarker et al., 2020b; Sarker and Oba, 2019b, 2019a, 2020). Amaranth leaves are especially rich in vitamin C. In *A. hypochondriacus*, the AHC11 genotype reached 184.77 mg/100 g FW. The overall average is 84.44 mg/100 g FW (Sarker and Oba, 2020). Compared with other leafy vegetables, amaranth leaves can contain up to 13 times more vitamin C than lettuce and about 3 times more than spinach on a fresh-weight basis (Sarker and Oba, 2019a). Vitamin C contributes to antioxidant capacity and acts synergistically with flavonoids and carotenoids (Sarker and Oba, 2019b).

Betalains, nitrogenous pigments characteristic of Amaranthaceae, are represented by betacyanins (red-violet) and betaxanthins (yellow-orange). Compounds such as amaranthine and isoamaranthine have shown antioxidant activity even higher than that of classic polyphenols (Matías et al., 2024; Thakur, Kumar, and Dhaliwal, 2021). In addition to flavonoids and carotenoids, the leaves contain phytosterols (mainly β-sitosterol), which have beneficial effects on lipid metabolism (Narwade and Pinto, 2018), as well as trace amounts of tocopherols, though at lower levels than in the seeds (Thakur, Kumar, and Dhaliwal, 2021). Phytosterols have been shown to reduce serum cholesterol and improve the cardiometabolic profile (Gupta et al., 2023; Matías et al., 2024; Narwade and Pinto, 2018; Stevanović et al., 2024). The concentration of these phytochemicals is highly dependent on the environment. In *A. tricolor*, salt stress significantly increases the levels of antioxidant compounds, demonstrating an adaptive response with relevant physiological and nutritional implications (Sarker et al., 2019).

#### 3.2.3 Antinutrients molecules

The leaves contain antinutrients such as oxalates and nitrates, and much lower levels of saponins and phytates than the seeds (Matías et al., 2024). In *A. tricolor*, soluble oxalate may reach 690 mg/100 g fresh weight, while total oxalate can exceed 1270 mg/100 g (Hricová et al., 2020). These concentrations are comparable to those in spinach and chard. They can decrease the bioavailability of calcium and magnesium if not properly processed (Kumar et al., 2024; Matías et al., 2024; Thakur, Kumar, Ahmed, et al., 2021). Traditional cooking methods like blanching, boiling, or stir-frying significantly reduce oxalate and nitrate levels (Hricová et al., 2020; Matías et al., 2024). This improves the bioavailability of minerals such as iron and calcium (Thakur, Kumar, Ahmed, et al., 2021). Unlike the seeds, the leaves contain minimal lectins or trypsin inhibitors. Therefore, their negative impact on protein digestion is very small (Matías et al., 2024).

Despite the presence of antinutrients, vitamin C’s reducing action enhances iron bioavailability, thereby improving its absorption (Narwade and Pinto, 2018). This explains why amaranth leaves are used in food programs aimed at combating iron deficiency anemia in vulnerable populations (Aderibigbe et al., 2022; Kumar et al., 2024; Sarker et al., 2020a).

#### 3.2.4 Nutritional and dietary relevance

Amaranth leaves possess a unique combination of high-density protein, essential minerals, vitamins, and phenolic compounds, making them a strategic food source for regions with high malnutrition and micronutrient deficiency rates and low dietary diversity (Zhu, 2023). Regular consumption contributes to bone health, anemia prevention, protection against oxidative stress, and immune system strengthening (Toimbayeva et al., 2025; Zhu, 2023). Furthermore, their capacity to accumulate carotenoids and betalains positions them as a relevant functional vegetable for the development of foods with antioxidant and nutraceutical properties (Matías et al., 2024; Stevanović et al., 2024). Furthermore, it offers agronomic advantages, including high productivity, stress tolerance, and the ability to grow in low-input agricultural systems, which increase its importance in the context of sustainable agriculture and food security (Akin Idowu et al., 2013; Matías et al., 2024; Zhu, 2023).

## 4 Applications and uses

Amaranth has long been valued for its nutritional density, but in the last decade it has emerged as a multifunctional crop with growing scientific interest. In addition to its use as a pseudocereal, new and diverse applications have appeared in fields such as human food, animal nutrition, sustainable agriculture, biotechnology, medicine, cosmetics, and pharmaceuticals. This positions amaranth as a strategic resource to address challenges in food security, health, and climate change. This grain is notable in human and functional nutrition for its high-quality protein, unsaturated lipids, and antioxidant bioactive compounds. This profile and its functional benefits support its use in various gluten-free products, including flours, breads, cookies, cereals, energy bars, beverages, sprouts, and supplements, improving traditional poorer flours as maize and rice (Coelho et al., 2018; Guevara et al., 2025). Processing methods, such as fermentation and instant controlled pressure drop (ICD), also enhance mineral bioavailability and boost antioxidant capacity (Das et al., 2025; Rodríguez-Castillo et al., 2025).

In human food and functional nutrition, amaranth stands out for its high-quality protein, unsaturated lipid content, and abundance of antioxidant bioactive compounds. These traits have led to its use in many gluten-free products, including flours, breads, cereals, energy bars, and beverages. Amaranth also improves the nutrition of traditional flours such as corn or rice flour (Coelho et al., 2018; Guevara et al., 2025). Modern processes like fermentation or instant controlled pressure drop (ICD) increase mineral bioavailability and antioxidant capacity in amaranth-based products (Das et al., 2025; Rodríguez-Castillo et al., 2025). Globally, food products derived from amaranth are growing steadily due to demand for functional and gluten-free ingredients. This trend encourages manufacturers to innovate and diversify amaranth-based offerings, reinforcing its role in health-focused and specialty foods. The global amaranth market exceeded USD 8 billion in 2024, with annual growth rates over 10% (Global Market Reports 2023–2024).

Its role in animal nutrition has also been widely studied. Due to its protein, fiber, and micronutrient content, amaranth has been incorporated into diets for ruminants, poultry, and rabbits. In poultry production, it can substitute up to 40% of conventional feed ingredients without impairing productivity (Hosseintabar-Ghasemabad et al., 2024). Trials in double-cropping systems have reported yields comparable to maize, making amaranth a sustainable feed alternative that enhances diet quality and productive efficiency in livestock systems (Das et al., 2025; Kaur et al., 2024; Manyelo et al., 2020). In sustainable agriculture, amaranth is considered strategic for its resilience to drought, marginal soils, and adverse climatic conditions. Integrating it into crop rotations and intercropping systems has been shown to have biofungicidal and nematicidal effects, reducing dependence on pesticides and promoting soil health. These practices contribute to yield stability and the conservation of agricultural biodiversity, which are crucial in the context of climate change (Das, 2016; Kaur et al., 2024; Parra-Cota et al., 2014).

Industrial interest in amaranth is also growing. Researchers have used extracts of *A. tricolor* to synthesize titanium dioxide (TiO_2_) nanoparticles with photocatalytic potential for the degradation of pollutants and their use as controlled-release carriers for bioactive compounds in food and pharmaceuticals (Rahmawati et al., 2024). In agricultural biotechnology, new applications are being explored for the production of biopolymers and biomaterials, as well as for studying genes associated with abiotic stress tolerance (Hadidi et al., 2024). Amaranth’s ability to form biodegradable films and starch blends for eco-friendly packaging has also been reported, one of the most relevant emerging applications within the circular economy. The capacity of amaranth to form biodegradable films and starch blends for eco-friendly packaging has been documented, representing a significant emerging application within the circular economy (Chandla et al., 2020; Karnwal et al., 2025). Additionally, the incorporation of natural pigments in innovative packaging materials is receiving increased attention. Recent studies have shown that anthocyanins derived from cabbage and sweet potato, when integrated into starch–PVA films, facilitate real-time assessment of shrimp freshness through distinct colour changes during spoilage (Zhang et al., 2020). This approach has been further developed by incorporating betalain pigments from red dragon fruit, periwinkle, beetroot, globe amaranth flowers, and red amaranth leaves into comparable film matrices, where shrimp spoilage is visually indicated by a colour transition from pink to yellow (Yao et al., 2021; Karnwal et al., 2025; Mahin et al., 2025). Collectively, these developments highlight the dual role of amaranth as both a structural material and a pigment source in the advancement of innovative, biodegradable packaging systems.

In the pharmacological and nutraceutical fields, amaranth contains bioactive compounds, including squalene, tocopherols, polyphenols, and other secondary metabolites. These have been shown to have antioxidant, anti-inflammatory, cholesterol-lowering, immunomodulatory, and cardioprotective effects (Adegbola et al., 2020; Chmelík et al., 2013; Liberal et al., 2016). Researchers have demonstrated that extracts exhibit antimicrobial activity against *Salmonella enterica* serovar Typhi and show potential as adjuvants in antifungal therapies against *Candida albicans* (De Vita et al., 2021). Its oil, rich in squalene and unsaturated fatty acids, is widely used in the cosmetics industry for its regenerative, emollient, and antioxidant properties, promoting skin hydration and cell renewal (Lacatusu et al., 2018; Mondor et al., 2020; Srivastava et al., 2021). In addition to these cosmetic applications, some species exhibit significant phytoremediation capabilities, including the accumulation of heavy metals such as lead, cadmium, and zinc (Cui et al., 2021; Zhou et al., 2024; Guo et al., 2020). Moreover, the allelopathic potential of the genus, mediated by flavonoids, alkaloids, and phenols, can inhibit the germination of crops such as wheat, soybeans, and corn by releasing these compounds into the soil, thereby disrupting crop establishment. This allelopathic effect presents both risks for crop production and opportunities for ecological weed management (Carvalho et al., 2019; Ganiee et al., 2024). Amaranth is important in biotechnology for its unique storage proteins, bioactivity, and stability. Notably, 11S globulins and other protein fractions are used to enhance crop nutrition, create functional foods, and produce bioactive recombinant proteins.

Building on this, several studies have shown that the *AmA1* gene can act as an effective biotechnological tool (Chakraborty et al., 2010). Specifically, its introduction into potatoes led to a quantifiable increase in the tubers’ protein content compared to non-modified potatoes, while its expression in maize enhanced the grain’s nutritional quality versus standard maize, without causing allergic reactions in animal models or consumers (Luna-Suárez et al., 2010; Chakraborty et al., 2010). These findings position amaranth proteins as safe candidates for biofortification programs.

Further highlighting their versatility, targeted molecular modifications have enhanced the nutraceutical value of globulins from the genus *Amaranthus*. The insertion of four repeats of the antihypertensive dipeptide Val-Tyr into the hypervariable region of the 11S globulin acid subunit resulted in a recombinant protein with ACE inhibitory activity measured as eight times greater than that of the unmodified, native 11S globulin (Luna-Suárez et al., 2010). This region, located on the surface of the cupin domain, facilitates proteolytic release of the active peptide, thereby increasing its efficacy as a potential antihypertensive agent. The Val-Tyr dipeptide also exhibits antiproliferative effects on vascular smooth muscle cells, expanding its potential biomedical applications (Matsui et al., 2005).

To translate these advances, modified proteins have been functionally expressed in heterologous systems such as *Escherichia coli* Origami (DE3) (Castro-Martínez et al., 2012). While these proteins initially accumulated in inclusion bodies, adjustments to temperature, agitation, and medium formulation enabled optimized production. This demonstrates the viability of amaranth as a molecular chassis for generating high-value-added bioactives (Tandang-Silvas et al., 2010).

**Figure 4:**
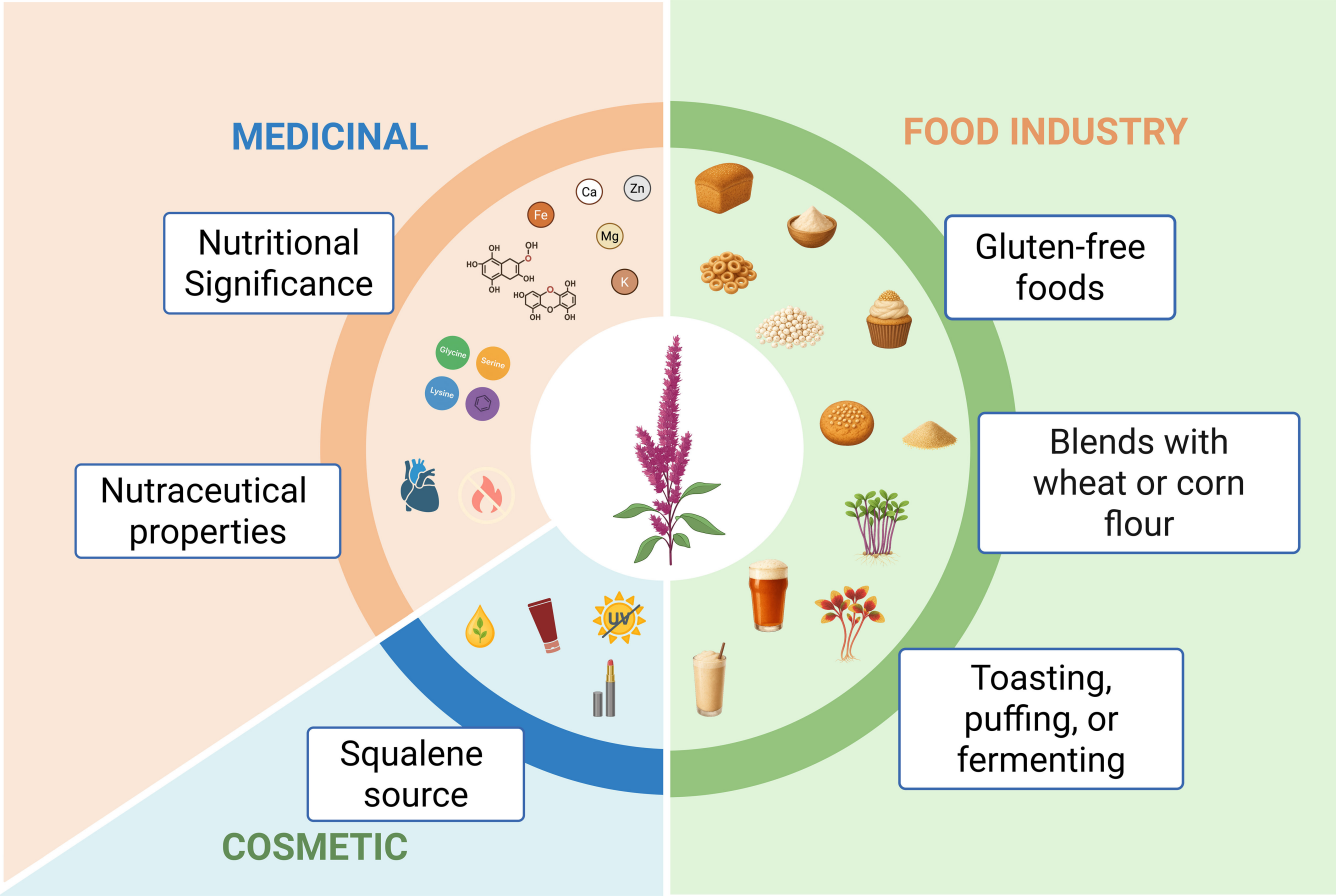
Overview of amaranth utilization. The crop provides value in cosmetics (squalene), nutraceuticals and medicinal applications, nutritional properties, and food industry uses, including flours, sprouts, and gluten-free products.

In addition to these functional attributes, unlike proteins from other crops—such as soybean beta-conglycinin—amaranth globulins have not shown allergenic activity, even when expressed in transgenic systems (Luna-Suárez et al., 2010; Cárdenas-Torres et al., 2019). This characteristic increases their value as a safe tool for food, pharmaceutical, and therapeutic applications. The versatility of amaranth is reflected in its wide range of applications. **Figure 4** summarizes the board uses of *Amaranthus* spp from food and feed to pharmaceutical, cosmetic, and industrial products. The global market for amaranth-derived products has shown sustained growth. In 2024, it reached USD 9.5 billion, with an estimated CAGR (Compound Annual Growth Rate) of 11.7%, driven by demand for functional foods, gluten-free products, and plant-based alternatives (Acumen Research and Consulting, 2024). Other analyses estimate values exceeding USD 16 billion by 2031 (Global Market Insights, 2024).

The global amaranth market is primarily driven by the Food and Beverage segment, which is the leading application. This segment is projected to grow at a CAGR of 10.81% from 2025 to 2032, fueled by increasing demand for healthy alternatives and gluten-free products (Maximize Market Research, s.f). Within this context, whole amaranth seed leads among products, capturing a 52.45% market share in 2024 (Maximize Market Research, s.f). Furthermore, amaranth oil reached a valuation of USD 6.1 billion in 2024 and is projected to grow at a CAGR of 17.7% through 2034 (GII Research, s.f). In addition, amaranth flour remains a key product and is the fastest-growing category due to its popularity in bakery and confectionery items (GII Research, s.f; Market Research Future, s.f). The growing presence of amaranth in the market suggests that consumers are highly receptive to this crop. Interest has been primarily motivated by the search for gluten-free products and flexible ingredients, which has encouraged its use in bread, pastries, and other food items (GII Research, s.f; Market Research Future, s.f; IMARC Group, s.f). Improvements in processing technologies have also enhanced its flavor and overall mouthfeel, helping consumers view it as an appealing and easy-to-use alternative (IMARC Group, s.f). The crop provides a significant income source for small-scale farmers, particularly women, due to its adaptability, short growing cycle, and growing demand in local and specialized markets (Kaur et al., 2024). Nevertheless, expansion is constrained by low mechanization, inconsistent quality standards, and limited integration into value chains.

## 5 Abiotic and biotic stress responses in *Amaranthus* spp

*Amaranthus* species are resilient to both abiotic and biotic environmental stresses. This resilience is due to various molecular, physiological, and biochemical mechanisms that support their adaptation to adverse conditions. Understanding these responses is key to utilizing amaranth as a stress-tolerant crop in marginal or changing environments.

### 5.1 Responses to abiotic stress

Abiotic stress refers to non-biological environmental factors—including drought, extreme temperatures, soil salinity, flooding, soil acidity (low pH), nutritional deficiencies, heavy metal toxicity, and ultraviolet radiation—that limit plant growth, survival, or reproduction (Lull et al., 2008; Greenway and Munns, 1980). These factors collectively threaten agriculture, as they can exacerbate yield loss when combined (Cramer et al., 2011; Bhargava and Srivastava, 2020). Climate change, by causing soil degradation, rising temperatures, and irregular precipitation, intensifies the issue. Thus, it is essential to study crops with high adaptive plasticity, which refers to their ability to acclimatize and thrive in various conditions. Underutilized species, such as *Amaranthus* spp., are strategic alternatives: they combine nutritional value with the natural ability to tolerate stress (Talabi et al., 2022). For a better understanding of the most relevant genes involved in abiotic stress responses in different *Amaranthus* spp., a summary in **Table 3A** is provided.

**Table 3A:** Genes and proteins involved in abiotic stress responses in *Amaranthus* spp.

Gene/protein	Functional type	Pathway/main function	*Amaranthus* species	Study type	Associated stress(es)	Observed phenotypic/physiological effect	References
CMO	Enzyme (choline monooxygenase)	Glycine betaine synthesis (osmoprotectant)	*A. tricolor*	Greenhouse/controlled solutions	Drought, salinity, heat	Increased osmolyte accumulation and improved turgor maintenance under stress	Ling et al., 2001; Meng et al., 2001
BADH	Enzyme (betaine aldehyde dehydrogenase)	Final step in glycine betaine biosynthesis	*A. tricolor*	Greenhouse/controlled solutions	Drought, salinity, heat	Higher betaine synthesis contributing to osmotic adjustment	Ling et al., 2001; Meng et al., 2001
Aquaporins	Membrane water channels	Water transport/osmoregulation	*Amaranthus* spp.	Transcriptomics/physiological assays	Drought, salinity	Improved hydraulic conductance and tissue water stability	Huerta-Ocampo et al., 2011, 2014
Nodulin-like proteins	Membrane/signaling proteins	Water and nutrient transport/signaling	*Amaranthus* spp.	Transcriptomics/in silico	Drought, salinity	Contribution to water homeostasis and root adjustment under stress	Huerta-Ocampo et al., 2011
DOF1	Transcription factor (DOF)	Regulation of C and N metabolism; responses to light and stress	*Amaranthus* spp.	Ortholog identification/gene expression	Drought, salinity, heat	Coordination of growth and stress response pathways, optimizing C use under limitation	Yanagisawa, 2000, 2001; Hu and Ma, 2006
MIF1	Mini zinc finger protein (MIF)	Regulator of development and hormonal responses	*Amaranthus* spp.	Ortholog identification/expression	Drought/multiple stresses	Modulates growth and architecture under stress; possible integration of hormonal signals	Hu and Ma, 2006; Huerta-Ocampo et al., 2014
AhNF-YC	Transcription factor (NF-Y, subunit C)	Regulation of drought-/ABA-responsive genes	*A. hypochondriacus*	Expression in amaranth; overexpression in *Arabidopsis*	Drought	Increased drought tolerance in *Arabidopsis* without severe growth penalties; breeding candidate	Délano-Frier et al., 2011; Palmeros-Suárez et al., 2015
AhDOF-AI	Transcription factor (DOF)	Regulation of salinity response and C metabolism	*A. hypochondriacus*	Overexpression in *Arabidopsis*	Salinity	Increased salt tolerance without affecting growth in the model system	Massange-Sánchez et al., 2016
Ah24	Amaranth-specific protein (putative function)	General response to salt stress	*A. cruentus*	Root proteomics (LC-MS/MS)	Salinity	Induced under NaCl; possible role in cellular protection and redox homeostasis	Huerta-Ocampo et al., 2014
Antioxidant enzymes (SOD, CAT, POD, GST)	Antioxidant defense enzymes	ROS detoxification, membrane protection	*A. cruentus, A. retroflexus*	Greenhouse/field; biochemistry	Heat, heavy metals, UV-B	Reduced lipid peroxidation, protein damage and maintained photosynthesis under stress	Netshimbupfe et al., 2022, 2023; Alsherif et al., 2022
PAL/CHS	Phenylpropanoid/flavonoid pathway enzymes	Phenol and flavonoid biosynthesis (UV shields, antioxidants)	*A. tricolor, A. retroflexus*	Greenhouse/UV-B; transcriptomics	UV-B radiation, high light	Increased flavonoids and betalains; improved photoprotection and UV-B tolerance	Adhikary et al., 2020; Sarker et al., 2019, 2020; Pandey and Agrawal, 2020
Betalain genes	Betalain biosynthesis enzymes	Pigments with antioxidant and photoprotective functions	*Amaranthus* spp.	Transcriptomic studies; targeted editing (CRISPR)	High light, UV, combined stress	Altered pigmentation and antioxidant capacity; potential targets for stress-tolerance editing	Liu et al., 2023

Several *Amaranthus* species are tolerant to drought, salinity, extreme temperatures, defoliation, high light intensity, and herbicide exposure (Brenner et al., 2000; Gaines et al., 2010; Rastogi and Shukla, 2013). These abilities are linked to traits such as C4 photosynthesis, indeterminate flowering, extensive lateral roots, osmolyte accumulation, high water-use efficiency, and expression of stress-responsive genes (Johnson and Henderson, 2002; Miller et al., 1984; Omamt et al., 2006). Sensitive plants such as *Arabidopsis thaliana* often show limited tolerance or growth when stress-responsive genes are overexpressed (increase in gene activity), causing abnormalities (Li et al., 2021; Sunkar et al., 2003). For this reason, stress-resilient species like amaranth are promising for finding useful genes for crop improvement.

#### 5.1.1 Drought stress

Drought is one of the most significant challenges for agriculture (Daryanto et al., 2016). *Amaranthus* uses physiological strategies such as reduced transpiration, increased water-use efficiency (WUE), osmotic adjustment via proline and raffinose oligosaccharides, and antioxidant accumulation (González-Rodríguez et al., 2019; Hasanuzzaman et al., 2023). At the whole-plant level, *A. cruentus* and *A. tricolor* exhibit greater transpiration efficiency under drought, which is reflected in increased yields (Jamalluddin et al., 2019). *A. hypochondriacus*, on the other hand, stands out for its osmotic adjustment and energy activation mediated by ABA and sugar scarcity signals (González-Rodríguez et al., 2019).

At the molecular level, drought tolerance in *Amaranthus* is linked to genes involved in osmoregulation, such as CMO and BADH, and to genes involved in water transport, such as aquaporins. Several transcription factors, including AhNF-YC and DOF family members, are also involved (Massange-Sanchez et al., 2015). Their roles are summarized in **Table 3A**. High expression of antioxidants and protective enzymes further contributes to tolerance, as seen in tolerant accessions of *A. tricolor* (Sarker et al., 2018).

#### 5.1.2 Salinity stress

Salinity tolerance differs among species and growth stages. *A. retroflexus* and *A. blitum* germinate efficiently at 25–200 mM NaCl, while *A.* sp*inosus* and *A. viridis* are less successful (Hao et al., 2017). In the field, *A. hypochondriacus* and *A. cruentus* show greater tolerance to saline irrigation than *A. tricolor* (Omamt et al., 2006). In *A. cruentus*, NaCl concentrations of 50–100 mM reduce growth but increase water-use efficiency by redistributing salt from the roots to the stems (Christophe et al., 2018; Luyckx et al., 2023; Gandonou et al., 2018). At the molecular level, proteins associated with carbohydrate metabolism, detoxification, and redox homeostasis stand out (Munns and Tester, 2008), as do regulatory factors such as AhDOF-AI, which improves salinity tolerance when overexpressed in Arabidopsis without penalizing growth (Massange-Sanchez et al., 2015; Massange-Sánchez et al., 2016). The complete set of differentially expressed proteins and their functions is summarized in **Table 3A**.

#### 5.1.3 Heat stress

Amaranth tolerates high temperatures better than many conventional crops. *A. cruentus* and *A.* sp*inosus* maintain greater PSII integrity and less photoinhibition under heat combined with drought, compared to *A. caudatus* and *A. hypochondriacus* (Netshimbupfe et al., 2022). Heat increases the levels of metabolites such as caffeic acid and rutin, thereby enhancing the nutraceutical value (Netshimbupfe et al., 2023). Antioxidant enzymes (SOD, catalase) and genes involved in RNA repair are also activated. Although the genus is thermotolerant, prolonged exposure to ~50°C drastically reduces yield (Reyes-Rosales et al., 2023). Key genes associated with this response are listed in **Table 3A**.

#### 5.1.4 Cold stress

As a C4 plant, amaranth is sensitive to cold: temperatures <15°C affect germination, photosynthesis, and yield, with symptoms such as chlorosis and necrosis (Gins, 2023; Gins et al., 2024). Empirical studies indicate that *A. cruentus* is more sensitive to cold than *A. hypochondriacus*, as evidenced by delayed flowering, reduced biomass, and greater drops in grain yield (Fuentes et al., 2020). Genes from families such as CBF/DREB and WRKY, along with cis-regulatory motifs such as EINL, participate in activating protective responses under cold conditions (Palmeros-Suárez et al., 2021), as summarized in **Table 3A**.

#### 5.1.5 Nutrient deficiencies

Although tolerant of marginal soils, amaranth reduces biomass and yield under N, P, or K deficiencies (Thanapornpoonpong, 2004; Acosta et al., 2014; Korres et al., 2017; Munene, 2017). The interactions between nutrition and endophytic microbiota are critical: macronutrient deficiencies favor pathogenic fungi and reduce beneficial bacteria, affecting quality and bioactive compounds (Lin et al., 2024). Strategies such as polyculture, biofertilizers, and mycorrhizae have been shown to improve nutrient uptake and crop quality (Vijayarengan et al., 2012; Mndzebele et al., 2020; Barba de la Rosa et al., 2022; Lin et al., 2024). The molecular mechanisms associated with nutrient deficiencies (e.g., genes regulating nutrient transport and antioxidant metabolism) are included in **Table 3A**.

#### 5.1.6 UV-B radiation stress

*Amaranthus* shows relative tolerance to UV-. This is due to the accumulation of flavonoids, phenylpropanoids, and betalains, which act as natural filters (Wang et al., 2007; Castrillón-Arbeláez and Délano-Frier, 2016; Portillo-Nava et al., 2021). UV-B increases the expression of genes such as PAL, CHS, and RCD. As a result, g melatonin, flavonoids, and phenols increase (Yavaş et al., 2020).

#### 5.1.7 Heavy metals toxicity

Amaranth is a hyperaccumulator, meaning it can absorb and store unusually high levels of cadmium (Cd), lead (Pb), and zinc (Zn). These metals are generally retained in the roots. However, some species translocate, or move, the metals to aerial tissues (Odiyi et al., 2019; Tőzsér et al., 2023; Zhou et al., 2024). Exposure to these metals increases oxidative stress—a harmful cellular condition caused by excess reactive oxygen species—which activates defense mechanisms. These include glutathione, phytochelatins, and metallothioneins (Alsherif et al., 2022), which help detoxify metals. Genotypic differences, or genetic variations, determine suitability for phytoextraction (using plants to remove contaminants) or safe production (Zhou et al., 2013; Yap et al., 2022). Mitigators (substances that reduce negative effects), such as zinc or melatonin, have been evaluated (Hunková et al., 2025; Madheshiya et al., 2025). Further details on genes and proteins involved in metal detoxification and transport are described in **Table 3A**. The following section discusses another important stress factor: mechanical stress and lodging.

#### 5.1.8 Mechanical stress and lodging

Although lodging is considered primarily an agronomic problem, its impact on yield makes it a key factor in management and improvement programs. *Amaranthus* spp., especially *A. hypochondriacus*, are susceptible to stem collapse due to heavy rains or strong winds, which reduces grain or biomass production and hinders harvesting (Romero Romano et al., 2021). In Japan (Kyushu), it has been documented that *A. hypochondriacus* forage crops suffer severe damage during typhoons. Direct seeding and inappropriate timing increase this risk, while delaying seeding or using “seed balls” helps to mitigate it (Zhong et al., 2024). Soil quality is also a determining factor; soils poor in organic matter, with low moisture retention capacity and weak root anchorage, increase susceptibility to lodging, although the incorporation of organic amendments, such as goat manure or crop residues, improves water retention, soil structure, and indirectly plant stability (Gabriela et al., 2016; Yadav and Yadav, 2024). Additionally, plants that are too tall, have thin stems, or are poorly established are more susceptible to falling over. However, practices such as transplanting, seed pretreatment, and sowing at dates adapted to the rainfall regime can reduce this problem (Henderson et al., 1998; Nurse et al., 2016; Zhong et al., 2024). However, because the molecular response to mechanical stress is less well understood than the response to other abiotic stresses, this represents a significant research gap.

### 5.2 Responses to biotic stress

Amaranth cultivation is affected by a wide range of biotic agents, including phytophagous insects, fungi, bacteria, phytoplasmas, viruses, and mites. These agents can seriously compromise grain and leaf production (Palmeros-Suárez et al., 2021). Most available information on pests comes from field surveys and farm observations in Africa, Asia, and the Americas. In contrast, the genetic basis of resistance is much less studied. Losses of up to 65% have been reported when no control measures are taken, especially from defoliating and seed-eating insects (García et al., 2011; Olsoni and Wilson, 1990). The phytophagous insect communities in *Amaranthus* are diverse, including defoliators, borers, miners, and sap-sucking insects from more than eight orders. Hemiptera, Coleoptera, Lepidoptera, and Thysanoptera are the most damaging, with documented impacts also caused by Diptera, Orthoptera, Hymenoptera, Neuroptera, and Mantodea (Ezeh et al., 2015; Thara et al., 2021; Salas-Araiza and Boradonenko, 2006; Seni, 2018). The severity of infestation depends on climate, agricultural practices, and crop species. Defoliators such as *Spoladea recurvalis* and *Psara basalis* are critical in Africa; *Liriomyza huidobrensis* reduces photosynthesis; *Myzus persicae* is significant in East Africa; and *Lygus lineolaris* leads to notable losses in the USA (Nampeera et al., 2019; Leland and McGuire, 2006; Wilson and Olson, 1990).

Among pathogens, Alternaria spp., Albugo bliti, Phomopsis, Pythium, Rhizoctonia, and Aspergillus flavus are the most relevant. Alternaria spp. cause leaf spots leading to reduced photosynthesis; Albugo bliti induces white rust affecting site and market value; Phomopsis triggers stem and leaf blight, leading to plant death; Pythium and Rhizoctonia cause damping-off and root rot, decreasing vigor and stand; Aspergillus flavus contaminates seeds, impacting food safety and marketability (Adebanjo and Ikotun, 1994; Keinath et al., 2003; Moran and Showler, 2007; Lee et al., 2020; Noelting et al., 2022; Zippora et al., 2022). Incidence increases under high humidity and abiotic stress conditions. Agroecological practices, such as polyculture with basil, have demonstrated reductions in lepidopteran infestations and increases in yield (Azandémè-Hounmalon et al., 2023). Integrated pest management (IPM) remains essential to the sustainability of amaranth cultivation. A summary of the most relevant genes involved in biotic stress responses in different *Amaranthus* spp. is presented in **Table 3B**.

**Table 3B:** Genes and proteins involved in biotic stress responses in *Amaranthus* spp.

Gene/protein	Functional type	Pathway/main function	*Amaranthus* species	Study type	Biotic agent/stimulus	Observed phenotypic effect/described function	References
AhSAG	Senescence-associated gene	Senescence signaling and damage response	*A. hypochondriacus*, *A. cruentus*	Greenhouse; mechanical and insect defoliation	Herbivory, mechanical damage	Stress marker and source–sink reorganization during and after defoliation	Castrillón-Arbeláez et al., 2012
AhSUT1	Sucrose transporter	Sugar transport; source–sink adjustment	*A. hypochondriacus*	Greenhouse; defoliation	Herbivory/foliar damage	Changes in sucrose transport; support for recovery of damaged tissues	Castrillón-Arbeláez et al., 2012
AhKTI	Kunitz-type trypsin inhibitor	Antiherbivore defense (inhibition of insect digestive proteases)	*A. hypochondriacus*	Greenhouse; induced expression	Defoliating lepidopterans	Increased inhibitory activity after attack; potential reduction of protein digestion in pests	Castrillón-Arbeláez et al., 2012
AhLOX2	Lipoxygenase 2	Oxylipin biosynthesis; JA pathway and damage signaling	*A. hypochondriacus*	Greenhouse; gene expression	Herbivory/wounding	Activation of the JA pathway and oxylipin-dependent defense	Castrillón-Arbeláez et al., 2012
ATI/AAI	Protease inhibitors (trypsin/amylase)	Antiherbivore defense in the insect gut	*Amaranthus* spp.	JA induction assays; bioassays	Lepidopteran attack/JA treatment	Increased inhibitory activity after attack or treatment; contribution to insect resistance	Délano-Frier et al., 2011; Sánchez-Hernández et al., 2004
Amaranth cystatins	Cysteine protease inhibitors	Antifungal activity against soilborne pathogens	*Amaranthus* spp.	*In vitro* fungal assays	*Fusarium oxysporum*, *Sclerotium cepivorum*, *Rhizoctonia solani*	Inhibition of fungal growth; potential use as resistance genes in other crops	Valdes-Rodriguez et al., 2010
PMEI	Pectin methylesterase inhibitor	Cell wall modification; pathogen resistance	*Amaranthus* spp.	Greenhouse; SAR models	*Pseudomonas syringae* pv. *tabaci*	Associated with induced resistance; reinforcement of cell wall integrity	Casarrubias-Castillo et al., 2014
PAL	Phenylalanine ammonia-lyase	Entry into the phenylpropanoid pathway; synthesis of phenolics/defenses	*Amaranthus* spp.	Greenhouse; defense inducers	Phytopathogenic bacteria	Increased phenolic compounds and lignification; involvement in systemic acquired resistance	Casarrubias-Castillo et al., 2014
ET/SA-related genes	Hormonal signaling regulators	Ethylene and salicylic acid pathways in bacterial resistance	*Amaranthus* spp.	SAR models; gene expression	*Clavibacter michiganensis* subsp. *michiganensis*	Activation of antimicrobial peptides and ET- and SA-dependent defense signals	Casarrubias-Castillo et al., 2014
AVP (Amaranth Antiviral Protein)	Antiviral protein purified from leaves	Inhibition of local viral infection	*A. tricolor*	*In vitro* assays and host plants	Sunnhemp rosette virus (SRV)	Reduction of local lesions in *Cyamopsis tetragonoloba*; potential antiviral tool	Roy et al., 2006
ACA (*Amaranthus caudatus* agglutinin)	Lectin/agglutinin	Interaction with insect membranes; defense	*A. caudatus*	Transgenic cotton transformation	Aphids (e.g., *Aphis gossypii*)	Transgenic cotton expressing ACA showed increased resistance to aphids	Wu et al., 2006
Betalain genes	Betacyanin/betaxanthin biosynthesis enzymes	Pigmentation, antioxidant activity, possible signaling	*A. hypochondriacus*	Transcriptomics; herbivory assays	Herbivory/foliar damage	Tissue- and genotype-dependent accumulation; possible role in signaling and defense	Casique-Arroyo et al., 2014
JA- and pathogen-responsive genes	Defense gene set (≈41% bacteria-related, 24% JA-related)	General response to infection and herbivory	*A. hypochondriacus*	Transcriptomics (microarrays/RNA-Seq)	Bacterial infection; herbivory	Broad defense reprogramming; identification of JA- and pathogen-regulated networks	Délano-Frier et al., 2011; Roy et al., 2006

#### 5.2.1 Molecular response to damage

Herbivory and pathogens trigger physiological and transcriptional changes, including adjustments in carbohydrate and lipid reserves and activation of jasmonate signaling pathways (Castrillón-Arbeláez et al., 2012; Palmeros-Suárez et al., 2021). Marker genes such as AhSAG, AhSUT1, AhKTI, and AhLOX2 are important for the regulation of senescence processes, sugar transport, and protease inhibition, as summarized in Table X. For pathogen defense, PAL, PMEI, antimicrobial peptides, and pathways activated by ethylene and cell wall oligomer-derived signals play significant roles (Casarrubias-Castillo et al., 2014). Additionally, antiviral proteins such as AVP from *A. tricolor* and genes from *A. caudatus* utilized in transgenic crops demonstrate the biotechnological potential of the genus for plant protection (Roy et al., 2006; Wu et al., 2006).

### 5.3 Knowledge gaps, gene transfer, and the potential of CRISPR editing in resistance to biotic stress

Research on stress responses in *Amaranthus* has advanced in recent years, yet we still know surprisingly little about how this crop deals with insects and disease (Priyadarsini et al., 2025; Eerapagula et al., 2025). Although amaranth is often described as resilient, it is not immune to biological threats. White rust (*Albugo bliti*) and *Alternaria* spp., for example, can significantly reduce yield when outbreaks occur (Priyadarsini et al., 2025; Keinath et al., 2003). One of the main reasons for this vulnerability is the limited availability of resistant cultivars. In addition, defense-related molecular markers are scarce, and progress in amaranth genetics has been slow (Joshi et al., 2018; Priyadarsini et al., 2025). Compared with other pseudocereals, breeders have fewer tools and less genetic information to work with (Sokolova et al., 2024; Yadav and Yadav, 2024). For that reason, CRISPR is attracting attention: by enabling precise modification of resistance genes, it offers a targeted approach to developing cultivars that can withstand biological threats and helps fill key knowledge gaps in amaranth’s molecular biology (Yadav and Yadav, 2024).

Another challenge is gene flow between cultivated amaranths and wild relatives (Yadav and Yadav, 2024; Gonçalves-Dias et al., 2023). Species in the genus cross easily, pushing unwanted traits into weedy populations. Herbicide resistance and stronger seed dispersal are two examples (Yadav and Yadav, 2024). This is most clear in *A. palmeri* and *A. retroflexus*, where hybrids often behave invasively. These issues complicate the release of transgenic lines, as transgenes can quickly spread to wild populations (Bansal and Kaur, 2025; Sokolova et al., 2024; Yadav and Yadav, 2024). Still, this gene flow can improve the crop. Introgressing reduced seed shattering from *A. powellii* into *A. hypochondriacus* and *A. cruentus* have produced varieties that keep more grain at harvest (Joshi et al., 2020; Sokolova et al., 2024). Nutritional genes like *AmA1* have also been moved to potatoes, maize, and wheat, increasing protein content (Joshi et al., 2020; Sokolova et al., 2024; Yadav and Yadav, 2024). These cases highlight the genus’s biotechnological value.

The arrival of CRISPR/Cas9 has changed the conversation about amaranth breeding (Priyadarsini et al., 2025). A reference genome for *A. hypochondriacus* is already available, and several genomic databases now support the identification of candidate genes linked to resistance, yield, or quality (Joshi et al., 2020; Priyadarsini et al., 2025; Singh et al., 2025). Using these resources, researchers recently produced the first stable edited lines of grain amaranth by targeting the betalain pathway, generating non-pigmented mutants (Priyadarsini et al., 2025; Vollmer et al., 2025). This demonstrates that the technique can work in the crop and may eventually allow the development of plants that tolerate fungi, insects, or other threats more effectively. However, the system still has practical limits. Regeneration *in vitro* is difficult, and Agrobacterium-mediated transformation rarely yields more than 30% stable plants (Joshi et al., 2020; Yaroshko et al., 2023).

CRISPR is also useful for understanding gene function, not just producing new varieties. Its capacity for precise genome editing enables researchers to experimentally determine the roles of individual genes in biotic stress responses, moving beyond sequence-based predictions (Joshi et al., 2020; Yadav and Yadav, 2024; Yaroshko et al., 2023; (Ajayi et al., 2024). Targeted editing, followed by testing in controlled conditions and in the field, could help map the main components of resistance and guide breeding strategies (Joshi et al., 2020). To reach this point, the field needs better transformation protocols, more genome and transcriptome resources focused on biotic stress, and multi-site evaluations to confirm that edited traits remain stable in real production environments (Joshi et al., 2020; Priyadarsini et al., 2025). In short, the situation combines promise with difficulty. Amaranth contains a large genetic reservoir, including many traits with agronomic or nutritional value that remain underexplored (Priyadarsini et al., 2025; Yadav and Yadav, 2024; Shukla et al., 2018). Yet its close relationship with weedy relatives means that any genetic intervention must be handled carefully to avoid unintended consequences (Bansal and Kaur, 2025). CRISPR enables highly targeted improvement, but only if technical and operational challenges are addressed (Bansal and Kaur, 2025; Priyadarsini et al., 2025). To transform amaranth from an underused resource into a resilient, high-value crop, the field requires sustained research, innovative technology, and rigorous, systematic evaluation (Bansal and Kaur, 2025; Priyadarsini et al., 2025).

## 6 Omics

Omics - including genomics (the study of DNA), transcriptomics (the study of RNA), proteomics (the study of proteins), and metabolomics (the study of small molecules and metabolites) - provide comprehensive molecular profiles of organisms. These approaches clarify the genetic layout, observable characteristics, and chemical processes within organisms. By monitoring DNA, RNA, proteins, and metabolites, omics facilitate the detection of changes driven by epigenetic (heritable changes not involving DNA sequence) and post-translational (modifications after protein synthesis) mechanisms; both can alter observable traits, or phenotypes. Genomics and transcriptomics describe how genes are regulated and expressed. Metabolomics measures the chemical products of these genes and the enzymes they produce. Taken together, these analyses yield a fast, detailed understanding of how genes and environmental factors interact (Maroli et al., 2015).

Building on these broad insights, recent omics approaches have transformed the study of *Amaranthus*. Researchers have uncovered the molecular basis for its metabolic plasticity, pigment and metabolite regulatory networks, stress response mechanisms, and high nutraceutical value (Gupta et al., 2023; Ma et al., 2021; Nemadodzi and Managa, 2024; Xue et al., 2025). However, despite these advances, omics resources for amaranth remain more limited than those available for crops such as rice, wheat, and maize (Clouse et al., 2016; Joshi et al., 2018; Schnable et al., 2009). This momentum has been further propelled by advancements in next-generation sequencing (NGS) technologies (Singh 2024), which have accelerated the acquisition of genomic, transcriptomic, and metabolomic datasets. However, omics approaches face several limitations. These include incomplete reference genomes, challenges with data integration, and difficulties translating large-scale datasets into biological insights (Lin et al., 2022; Sedeek et al., 2025; Vollmer et al., 2025). Consequently, *Amaranthus* has emerged as a model system for integrative studies (United States Department of Agriculture, National Institute of Food and Agriculture, 2025). Multi-omics analyses have revealed genomic duplications, gene family expansions, biosynthetic gene clusters, pigment regulatory networks, and stress tolerance mechanisms (Ma et al., 2021; Sedeek et al., 2025; Singh et al., 2023; Wang, 2023). Researchers have also catalogued hundreds of metabolites across diverse tissues and environmental conditions. These include amino acids, fatty acids, phenolic compounds, saponins, and osmoprotectants (Gupta et al., 2023; Nemadodzi and Managa, 2024). Collectively, these advances inform molecular design of functional foods, genetic improvement strategies, and valorization of amaranth as a nutraceutical crop (Araujo-León et al., 2024; Gupta et al., 2023). As an overview of omics studies conducted in *Amaranthus* spp, the **Table 4** is presented with the aim of highlighting the most relevant advances on this field.

**Table 4:** Overview of omics studies conducted in *Amaranthus* spp.

Omics study type	Species/variety	Tissue/organ	Approach/platform	Main finding	References
Genomics	*Amaranthus powellii*, *A. bouchonii* and 25 additional species	Leaves (chloroplast genomes)	Chloroplast genome assembly and comparison; phylogeny	Clear phylogenetic separation between *A. powellii* and *A. bouchonii*; variable markers identified	(Han et al., 2025)
Genomics	*Amaranthus cruentus*	Whole genome (entire plant)	Short- and long-read sequencing, Hi-C	Chromosome-level assembly; identification of biosynthetic clusters and whole-genome duplication	(Ma et al., 2021)
Genomics	*Amaranthus* spp. (multiple species)	Seeds/germplasm bank plants	ddRAD, GWAS, SNP calling	Genetic differentiation; genes associated with flowering (AGL20/SOC1)	(Lin et al., 2022)
Genomics	Dioecious *Amaranthus* species	Chloroplast	Plastome assembly and phylogeny	Monophyly of *Acnida*; low divergence between *A. palmeri* and *A. watsonii*	(Raiyemo and Tranel, 2023)
Genomics	Five species: *A. hypochondriacus, A. tuberculatus, A. hybridus, A. palmeri, A. cruentus*	Genomes	Repeat analysis, ortholog identification, phylogeny	Abundant LTRs (14–25%); clade separation; relevance for breeding	(Singh 2024)
Genomics	*Amaranthus tricolor*	Leaves	R2R3-MYB family identification; qRT-PCR; VIGS/yeast/dual-luciferase	*AtrMYB72* activates *CYP76AD1* → increased betalain accumulation; 73 MYBs identified in genome	(Xue et al., 2025)
Genomics	*A. palmeri, A. retroflexus, A. hybridus*	Chromosome-level genomes	PacBio, Hi-C, BioNano	Sex-determining region (~2.84 Mb) with 37 genes; *RF1* and *TLC* upregulated in males; EPSPS eccDNA detected	(Raiyemo et al., 2025)
Genomics	*A. tricolor*	Leaves (red vs green)	PacBio HiFi + Nanopore + Hi-C	Ancestral WGD; co-expression of *DODAα1* and *CYP76ADα1* linked to betalain biosynthesis	(Wang et al., 2023)
Metabolomics	*A. hybridus, A. blitum, A. caudatus*	Aerial parts/leaves	LC-MS/MS + PCA	41 compounds identified; chemotaxonomic markers; first report of N-coumaroyl-L-tryptophan and adenosine	(Abdel-Moez et al., 2023)
Metabolomics + Genomics	Five cultivars: CF, ET, GG, HR, NM (*A. caudatus, A. cruentus, A. hypochondriacus*)	Grain and leaves; whole genome	UPLC-MS/MS, PacBio Sequel II WGS, Iso-Seq	420–426 metabolites detected; nutritional differences; reference genomes assembled	(Sedeek et al., 2025)
Metabolomics	*Amaranthus cruentus*	Grain (two stages: immature vs mature)	Metabolomics + nutritional chemistry	Mature grain contained higher mineral and secondary metabolite levels	(Manyelo et al., 2022)
Metabolomics	*Amaranthus palmeri*	Resistant and susceptible biotypes	GC-MS, LC-MS/MS; enzymatic activity	Antioxidants complement glyphosate resistance; metabolic recovery	(Maroli et al., 2015)
Metabolomics	*A. graecizans* (green) and *A. cruentus* (red)	Leaves (field vs greenhouse)	¹H-NMR	Differences driven by cultivation system; allantoin detected only in greenhouse-grown green plants	(Nemadodzi and Managa, 2024)
Metabolomics	*Amaranthus cruentus* (red accession)	Leaves, inflorescences	HPLC-UV-DAD, UV-Vis; *in vitro, in vivo, ex vivo* assays; docking	High flavonoid content in leaves and betacyanins in inflorescences; antidiabetic and antihypertensive activity	(Araujo-León et al., 2024)
Metabolomics	*A. cruentus, A. hybridus* (wild vs cultivated)	Leaves	¹H-NMR + LC-MS	Higher sugar levels in cultivated types; rutin and saponins detected; distinct metabolic profiles	(Nkobole & Prinsloo, 2021)
Metabolomics	*Amaranthus tricolor*	Hypocotyls	LC-MS/MS (blue light vs darkness)	37 differential metabolites; increased lipids under blue light; anthocyanins detected	(Xiao et al., 2022)
Proteomics	*A. hypochondriacus*	Leaves (water stress)	2-DE, LC/ESI-MS/MS	Regulation of chloroplast and mitochondrial proteins; chaperonins increased, Rubisco decreased under drought	(Huerta-Ocampo et al., 2009)
Proteomics	*A. hybridus*	Roots (Cd stress)	2-DE + MALDI-TOF/TOF; qPCR	28 differential proteins; increased energy and defense metabolism; Cd tolerance via root metabolic redirection	(Jin et al., 2016)
Proteomics	*A. cruentus*	Seed	2-DE + LC-MS/MS	Identification of LEA proteins and defense/respiration proteins; LEA cloning	(Maldonado-Cervantes et al., 2014)
Proteomics	16 *Amaranthus* spp.	Seeds	MALDI-TOF-MS biotyping	Protein spectra enabled rapid species/regulatory identification; 87–100% accuracy	(Murphy et al., 2023)
Proteomics	*A. hypochondriacus*	Seeds	In silico + MS + simulated digestion	Six CRPs characterized; digestion-resistant peptides with bioactive potential	(Moyer et al., 2022)
Transcriptomics	*A. palmeri* (tolerant vs susceptible)	Leaves, 24 HAT after glufosinate	Illumina RNA-Seq	567 DEGs; tolerant plants showed higher ABC, GST, NAC, CERK1, HSPs and CYP expression; pre-existing tolerance	(Salas-Perez et al., 2018)
Transcriptomics + Genomics	*A. hypochondriacus* + resequencing of *A. caudatus, A. cruentus, A. hybridus*	Whole genome, multiple tissues	WGS ALLPATHS-LG, BioNano, RNA-Seq	377-Mb genome assembly; 23,059 genes; SNPs support *A. hybridus* as progenitor; synteny with beet	(Clouse et al., 2016)
Transcriptomics + Genomics	*A. caudatus* vs Mexican hybrid	Seeds at three stages	Genome-seq; ω-3 and oxylipin pathway gene expression	Kiwicha accumulated more linoleic/linolenic acids; higher Δ6-desaturases; Mexican hybrid showed higher lipoxygenase and jasmonate levels	(Kanbar et al., 2023)
Transcriptomics + Metabolomics	*Amaranthus retroflexus* (resistant and susceptible)	Leaves	RNA-Seq (NovaSeq 6000), qRT-PCR, LC-MS/OPLS-DA	979 DEGs in resistant vs control, 15,731 in susceptible plants; amino acid pathways linked to fomesafen resistance	(Song et al., 2025)
Transcriptomics + Metabolomics	*Amaranthus tricolor*	Hypocotyls under blue light vs darkness	RNA-Seq + pigment quantification	Blue light increased betalains and flavonoids; upregulation of *CYP76AD, DODA*, and *DELLA*; short red hypocotyls	(Liu et al., 2023)
Transcriptomics + Proteomics + Metabolomics	*A. palmeri* (GR vs GS)	Leaves, 24 HAT after glyphosate	RNA-Seq, LC-MS/MS, metabolomics	GR plants maintained homeostasis; GS plants showed collapse of primary metabolism; increased glutathione in GR	(Sandhu et al., 2025)
Population genomics (GBS)	*A. hypochondriacus, A. caudatus, A. cruentus, A. hybridus, A. quitensis*	Seeds	GBS, gene flow analysis	Gene flow reduced genetic load; hybrid incompatibilities identified	(Gonçalves-Dias et al., 2023)
Genomics + Phenotyping	*A. caudatus, A. hybridus, A. quitensis*	Seeds	GBS + phenotyping	Incomplete domestication; absence of a strong bottleneck	(Stetter and Schmid, 2017)
Phylogeny + Genome size	35 *Amaranthus* spp.	Multiple accessions	GBS, flow cytometry	Subgenera supported; recent polyploidy in two lineages	(Stetter and Schmid, 2017)

### 6.1 Genomic

The first major genomic milestone in *Amaranthus* was achieved with the sequencing *of A. hypochondriacus*. In 2016, researchers published the first high-quality draft of the ‘Plainsman’ grain amaranth genome, revealing a 377 Mb assembly across 3518 scaffolds (N50: 371 kb). Key findings included an estimated total genome size of 466 Mb, with 48% consisting of repetitive sequences (mainly retrotransposons), and the annotation of 23,059 protein-coding genes. A significant subsequent advance was the construction of a *de novo* physical map (~340 Mb) using BioNano Genomics, which enabled a hybrid assembly that nearly doubled the N50 to 697 kb and greatly improved genome continuity (Clouse et al., 2016).

In a second phase, researchers used these resources to resequence cultivated accessions of *A. hypochondriacus*, *A. caudatus, A. cruentus*, and *A. hybridus*. This led to the identification of 7,495,570 SNPs, supplemented by 41,931 SNPs from GBS analysis. These findings clarified population structure and confirmed *A. hybridus* as the progenitor of clouse amaranth. Furthermore, Ks analysis indicated that the most recent whole-genome duplication (WGD) shared by *A. cruentus* and *A. hypochondriacus* occurred about 18.4–34 million years ago (Clouse et al., 2016). Subsequent comparative studies revealed both chromosome losses and fusions, with the unique fission of chromosome 2 in *A. cruentus* explaining its chromosome number (n = 17) compared to n = 16 in *A. hypochondriacus* (Clouse et al., 2016; Ma et al., 2021). Sequencing chloroplast genomes across several *Amaranthus* species yielded key insights into phylogenetic relationships, genetic diversity, and patterns of domestication (Adhikary et al., 2021; Han et al., 2025), while complementary synteny analyses with Beta vulgaris provided additional insights into the timing and mechanisms of polyploidy events and chromosome rearrangements (Clouse et al., 2016).

In a more recent phase, complete chromosomal assemblies were achieved for *A. cruentus* (≈365–371 Mb) (Ma et al., 2021) and *A. tricolor* (520 MB) (Wang et al., 2023). These efforts identified gene family expansions and duplications and revealed the organization of biosynthetic clusters linked to pigments and antioxidants (Ma et al., 2021). Building on such advances, genomic data from weedy species like *A. tuberculatus*, *A. hybridus*, and *A. palmeri* enabled studies on the evolution of dioecy and herbicide resistance (Montgomery et al., 2020; Raiyemo et al., 2025). Most recently, the publication of high-quality reference genomes (465–483 Mb) for five agronomically important cultivars—including *A. caudatus* ‘Coral Fountain’ and *A. cruentus* ‘Golden Giant’—integrated genomic and metabolomic data, further deepening understanding of gene families tied to nutritional and nutraceutical properties of the grain (Sedeek et al., 2025).

### 6.2 Transcriptomics

The first transcriptomic studies in *Amaranthus* focused on *A. hypochondriacus* because no complete reference genome was available at the time. In these studies, researchers assembled a *de novo* transcriptome of 66,370 contigs from eight tissue libraries exposed to abiotic stress. This resource was used as the primary basis for gene annotation (Adhikary et al., 2021). Notably, 97% of RNA-Seq reads mapped back to this assembly. Importantly, functional analysis showed the transcriptome included nearly all enzymes for key metabolic pathways, specifically 21 of 22 enzymes involved in aromatic amino acid synthesis and all eight enzymes required for branched-chain amino acid (Clouse et al., 2016). biosynthesis, highlighting the assembly’s completeness and utility for future studies.

Later, transcriptomic studies in *A. hypochondriacus* used the pyrosequencing platform to generate 21,207 contigs from six cDNA libraries. These studies identified 1,971 transcripts whose expression changed in response to water stress, salinity, herbivory, and bacterial infection (Adhikary et al., 2021; Délano-Frier et al., 2004; Singh 2024). These findings facilitated the mapping of gene networks controlling responses to abiotic and biotic stress (Castrillón-Arbeláez and Délano-Frier, 2016). Separately, researchers compiled over 31.7 Gbp of transcriptomic data from various tissues and developmental stages of *A. hypochondriacus*, thereby improving the accuracy of functional annotation and aiding the identification of candidate genes (Clouse et al., 2016).

Transcriptomics has also been used in weedy and cultivated species for specific functional studies. In *A. retroflexus*, transcriptomics and metabolomics were combined to examine resistance to the herbicide fomesafen. This work showed the role of amino acid pathways in tolerance mechanisms (Song et al., 2025). In *A. palmeri*, researchers identified overexpression of cytochrome P450 and ABC transporters associated with glufosinate resistance (Salas-Perez et al., 2019).In *A. tuberculatus*, candidate herbicide target genes have been identified, including HPPD, GS, EPSPS, and ALS (Clouse et al., 2016; Salas-Perez et al., 2019). Transcriptomics has also explored pigmentation. *In A. tricolor*, RNA-Seq characterized betalain and flavonoid biosynthesis in red and green leaves (Liu et al., 2023). It identified many differentially expressed unigenes and revealed pathways regulated by blue light versus darkness (Liu et al., 2023). More recently, studies in *A. caudatus* showed that cold during seed development regulates genes in the ω-3 fatty acid pathway and jasmonate signaling (Kanbar et al., 2023). These studies together define regulatory networks essential for pigmentation, secondary metabolism, stress adaptation, and nutritional quality (Castrillón-Arbeláez and Délano-Frier, 2016; Kanbar et al., 2023).

### 6.3 Proteomics and peptidomics

Proteomics in *Amaranthus* was first used to study the response to abiotic stress (orchard). For example, a pioneering study on *A. hypochondriacus* used comparative proteomics (2-DE and LC/ESI-MS/MS) to analyze drought-treated leaves. This analysis identified upregulated proteins, mainly chloroplast chaperonins such as Chaperonin-60 α and β. It also revealed downregulated proteins. These included the large subunit of Rubisco, the cytochrome b6f complex, oxygen evolution complexes (OECs), and mitochondrial ascorbate peroxidase ((Huerta-Ocampo et al., 2009). Taken together, these results highlight the central role of chloroplasts and mitochondria in adaptation to water stress. In a later stage, proteomics of *A. cruentus* (cv. Amaranteca) seeds resolved approximately 400 proteins using 2-DE. Among these were proteins linked to stress response, defense, metabolism, respiration, and redox processes (Maldonado-Cervantes et al., 2014; Toimbayeva et al., 2025). Notably, one key finding was the identification, cloning, and characterization of a Group 5 LEA protein (AcLEA). This protein is involved in desiccation protection during late embryogenesis. It is considered a novel member of this family (Toimbayeva et al., 2025).

Subsequently, protein biotyping strategies were developed using MALDI-TOF-MS. This approach generated protein fingerprints from seeds of 15 *Amaranthus* species, enabling grouping and assessment of genetic proximity. However, closely related species such as *A. powellii* and *A. hybridus* showed overlapping patterns. This overlap probably results from gene flow. In a shift towards functional bioactive molecules, more recent peptidomic studies in *A. tricolor* aerial tissue identified multiple classes of antimicrobial peptides (AMPs). These peptides show biotechnological and nutraceutical potential (Moyer et al., 2021). Furthermore, targeted analysis of storage proteins has characterized peptides derived from 11S globulin and albumin. These peptides show antioxidant, antihypertensive, and antimicrobial activities (Moyer et al., 2021; Quiroga et al., 2007, 2017). Taken together, these results reinforce the role of amaranth proteins and peptides as a source of bioactive molecules of nutraceutical interest.

### 6.4 Metabolomics

The application of metabolomics in *Amaranthus* began with non-targeted studies focused on phytochemical and chemotaxonomic characterization. For instance, edible species such as *A. hybridus*, *A. blitum*, and *A. caudatus* were profiled using LC-MS/MS. These studies identified 41 compounds—mainly amino acids, flavonoid glycosides, and hydroxycinnamoyl amides. Isorhamnetin and tricine glycosides, exclusive to *A. caudatus*, were described as chemotaxonomic markers (Abdel-Moez et al., 2023). Notably, these studies reported, for the first time in Amaranthaceae, metabolites such as adenosine and N-coumaroyl-L-tryptophan (Abdel-Moez et al., 2023).

Building on these initial applications, metabolomics was also applied to the study of stress response and nutritional quality. Metabolomic profiles enabled differentiation of glyphosate-resistant and susceptible biotypes of *A. palmeri*. This highlighted the roles of specific metabolic pathways in herbicide resistance (Kaur et al., 2024; Maroli et al., 2015). In red amaranth (*A. tricolor*) leaves, 444 metabolites were identified, with 37 showing significant variations under blue light compared to darkness. Increases were observed in 15 lipids and a decrease in several amino acids. Additionally, researchers detected components of the anthocyanin pathway, such as cyanidin chloride and anthocyanin 3-O-β-D-glucoside (Liu et al., 2023; Xiao et al., 2022).

More recently, continued advancements in metabolomics using LC-MS/MS, GC-MS, and NMR have further consolidated the nutraceutical profile of amaranth (Abdel-Moez et al., 2023; Gupta et al., 2023). For example, high-throughput, non-targeted profiling (UPLC-MS/MS) in five cultivars of *A. caudatus*, *A. cruentus*, and *A. hypochondriacus* identified 426 metabolites in grains and 420 in leaves. Significant variations were found in sulfur-containing amino acids, vitamins, chlorogenic acids, oxalates, and raffinose oligosaccharides (RFOs) (Sedeek et al., 2025). Additionally, in *A. hypochondriacus* seeds, GC-MS and NMR studies highlighted high levels of squalene, lysine, unsaturated fatty acids, stigmasterol, and other bioactive lipids (Gupta et al., 2023). Environmental metabolomics by 1H-NMR has also shown that *A. graecizans* and *A. cruentus* alter the accumulation of trehalose, betaine, allantoin, and other osmoprotectants depending on the cultivation system. These included comparisons between open field and greenhouse systems (Nemadodzi and Managa, 2024).

## 7 Breeding and genetic transformation

**Figure 5:**
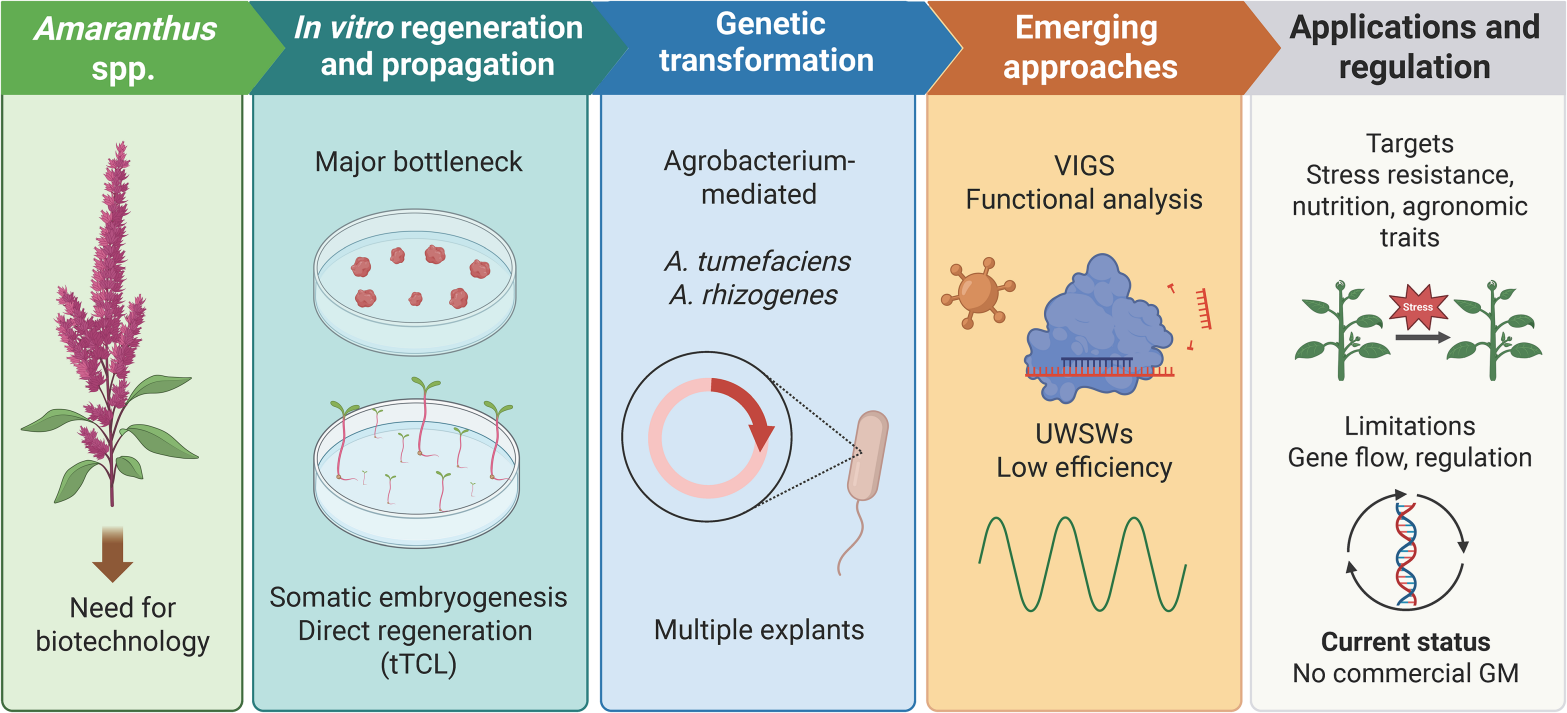
Different scientific approaches that can be applied to improve the cultivation and genetic transformation of *Amaranthus* spp. From the need of using biotechnology in *Amaranthus* ssp., *in vitro* methods to regenerate and propagate *Amaranthus* and using species of Agrobacterium tumefaciens or A. rhizogenes, different explants can be used for genetic transformation. Moreover, emerging approaches such as VIGS can be also applied. Finally, new GM or edited *Amaranthus* varieties could be generated to improve the agricultural and commercial traits of this pseudocereal.

Even with its many reported benefits and a long history of cultivation, cultivated *Amaranthus* spp. remain underutilized, having received little attention in terms of genetic improvement and systematic breeding. Moreover, the molecular mechanisms underlying defense against both biotic and abiotic stresses are still incompletely understood, and many desirable traits remain understudied. Although their nutritional properties are well documented, substantial opportunities for enhancement persist, and these can be addressed through biotechnological approaches. In this context, genetic transformation stands out as a promising strategy to improve key agronomic and quality traits, and greater effort should be directed toward generating more stable phenotypes. The different scientific approaches that can be applied to improve the cultivation and genetic transformation of *Amaranthus* spp. in the present and in the future are summarized in **Figure 5**.

### 7.1 *In vitro* regeneration as a prerequisite

The establishment of efficient *in vitro* regeneration protocols is a critical prerequisite for successful genetic transformation. Although *Amaranthus* is generally considered a recalcitrant genus in this regard, several advances have been made to improve regeneration efficiency (Castellanos-Arévalo et al., 2020; Wolosik and Markowska, 2019; Yaroshko et al., 2019). The genus division into grain and leafy amaranths (Wolosik and Markowska, 2019), with grain types showing higher recalcitrance to indirect transformation methods than their leafy counterparts (Castellanos-Arévalo et al., 2021), despite multiple transformation attempts (Jofre-Garfias et al., 1997; Yaroshko et al., 2019).

Somatic embryogenesis is the most common technique because it regenerates whole plants from a single cell, which is essential for the production of stable transformed lines (Bennici et al., 1992, 1997; Flores et al., 1982; Jan et al., 2020; Pal et al., 2013; Castellanos-Arévalo et al., 2021). Researchers have used leaf discs, hypocotyls, epicotyls, and nodes as explants to induce regeneration, with results varying by genotype (Flores et al., 1982; Bennici et al., 1992; Jan et al., 2020).

However, to overcome the limitations of regeneration associated with these methods, the cross-sectional thin-layer technique (tTCL) has been used to successfully regenerate *A. edulis* (also known as *A. caudatus*), a species widely considered one of the most recalcitrant. Complete plant regeneration was achieved using tTCLs excised from the apical or subapical zones and cultured in media supplemented with diphenylurea derivatives (TDZ and CPPU), auxins, and cytokinins, without passing through the callus phase (Tran Thanh Van, 1973; van Le et al., 1998). This approach is particularly valuable given the difficulties in regenerating amaranth after transformation (Bennici et al., 1997).

### 7.2 Propagation of regenerated plantlets

In addition to transformation, *in vitro* propagation of transgenic amaranth plantlets is necessary to obtain stable lines. Although limited information is available, Jan et al. (2020) successfully propagated *A. viridis* using nodal explants in a medium containing BA and NAA to induce shoot and root formation. This protocol also addressed hyperhydricity by maintaining the pH between 5.8 and 6.0 and adjusting the concentration of the solidifying agent (0.6–0.7%). Other studies have confirmed the effectiveness of strong cytokinins, such as TDZ, in enhancing shoot proliferation in genotypes with low shoot multiplication capacity (Gajdošová et al., 2013). Nevertheless, amaranth regeneration remains strongly dependent on genotype, explant source, and growth regulator combinations (Bennici et al., 1997).

### 7.3 Genetic transformation techniques in *Amaranthus*

**Figure 6:**
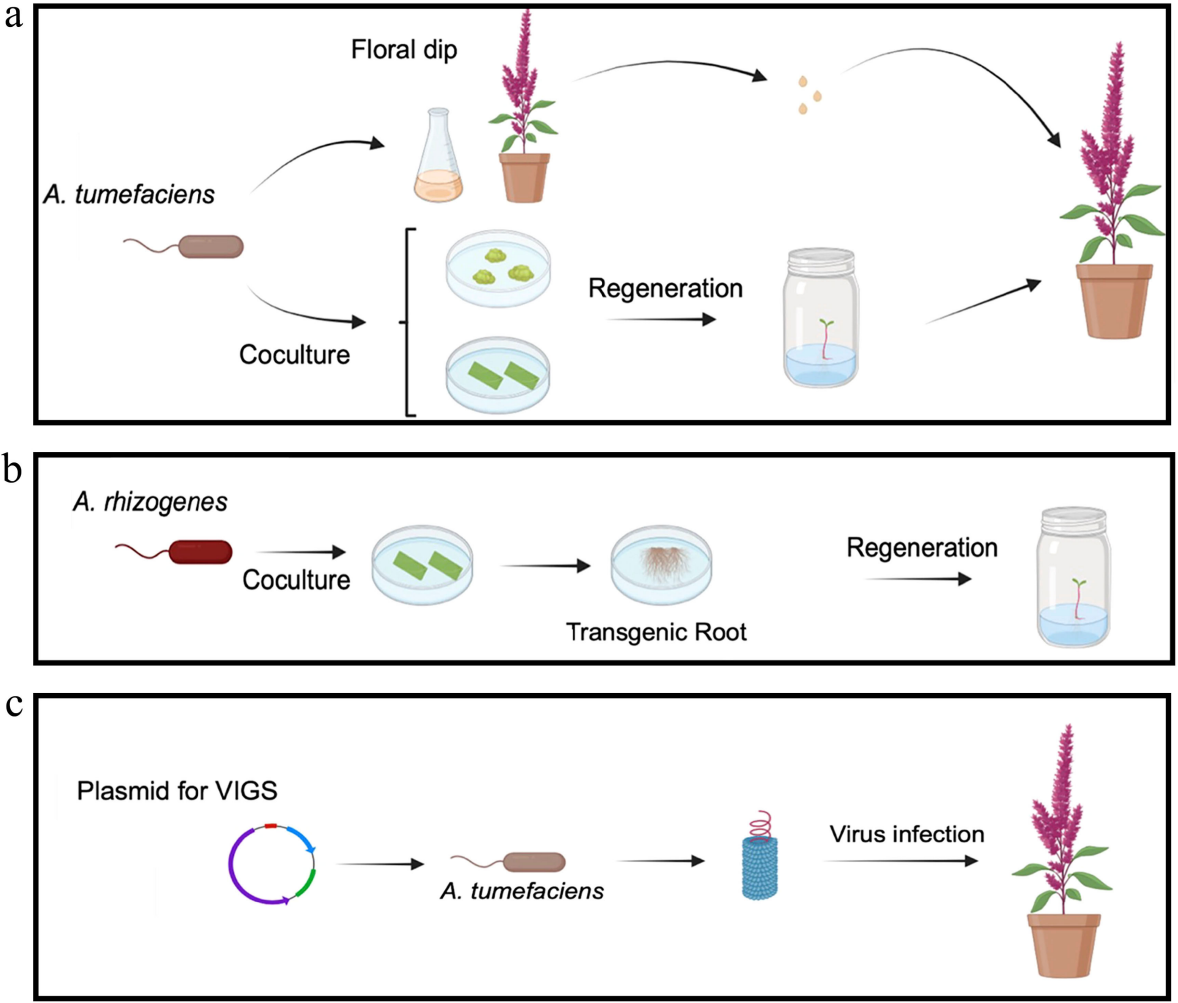
Overview of genetic transformation methods in *Amaranthus*. **(a)***Agrobacterium tumefaciens*-mediated transformation by floral dip or tissue culture regeneration; **(b)***Agrobacterium rhizogenes*-mediated root transformation; and **(c)** virus-induced gene silencing (VIGS) as a transient expression system.

Improving defensive traits in amaranth—such as resistance to fungal pathogens (*Aspergillus*, *Fusarium*) and insect pests like aphids—can be achieved through genetic transformation. In recent years, several protocols have been developed using *Agrobacterium tumefaciens* and *Agrobacterium rhizogenes* as gene delivery systems (**Figure 6**). Historically, amaranth was considered poorly susceptible to *Agrobacterium* infection (De Cleene and De Ley, 1976), but Jofre-Garfias et al. (1997) demonstrated successful transformation of *A. hypochondriacus* using the oncogenic strains pTiC58 and A281 on internode and hypocotyl explants. Since then, various explants have been explored. Pal et al. (2013) reported that epicotyl explants from *A. tricolor* had higher agroinfiltration efficiency than hypocotyls, with strain EHA105 being the most effective. Embryonic tissue has also been used as an explant, with embryos excised and co-cultured with *A. tumefaciens* strain pTiB6S3, resulting in transgenic plants with stable T-DNA insertion (Jofre-Garfias et al., 1997). Additionally, the floral dip technique has been successfully applied in *A. caudatus*, *A. retroflexus*, and a hybrid between *A. caudatus* and *A. paniculatus* (Kuluev et al., 2017; Yaroshko et al., 2019). Transformation with *A. rhizogenes* enables the generation of transgenic roots, from which whole plants have been regenerated in *A. hypochondriacus*, *A. hybridus*, and *A. tricolor* (Castellanos-Arévalo et al., 2020; Swain et al., 2010). This method also allows the establishment of transgenic root cultures, which are useful for producing secondary metabolites. While *Agrobacterium*-mediated transformation is now feasible in amaranth, its success remains highly dependent on the bacterial strain, plant species, explant type, and co-culture conditions.

### 7.4 Emerging transformation approaches

New strategies such as virus-induced gene silencing (VIGS) have recently been tested in *A. tricolor*. Adhikary et al. (2019) used a tobacco rattle virus (TRV)-based system to silence the *AtriCYP76AD1* gene, involved in betalain biosynthesis. Silenced plants exhibited loss of red pigmentation in the leaves, confirming the functionality of the system, although the phenotype was not inherited in the seed coat of progeny. Another emerging technique is the use of Underwater Direct Sound Waves (UWSWs), which enhance plasma membrane permeability to facilitate DNA uptake. Nava-Sandoval (2018) applied this method to *A. hypochondriacus* seeds, producing transgenic plants, albeit at a low transformation efficiency (<3.75%). The resulting plants may have been chimeric, and further optimization is required. Although *Amaranthus* has long been considered recalcitrant to regeneration and genetic transformation, recent developments—particularly in somatic embryogenesis, *Agrobacterium*-mediated protocols, and novel techniques such as VIGS and UWSWs—indicate growing potential for genetic improvement. These advances open new opportunities for molecular breeding aimed at enhancing stress tolerance, nutritional content, and agronomic performance in this resilient crop.

### 7.5 Regulatory and biosafety considerations

Regulatory oversight of genetically modified (GM) cultivars focuses on evaluating their safety and ensuring compliance with international standards, including those established by the Codex Alimentarius (Goodman, 2014). The principle of substantial equivalence is applied to determine whether foods derived from GM events are as safe as their conventional counterparts (Goodman, 2014). These evaluations address the potential allergenicity of newly introduced proteins, assess toxicity and antinutrient levels, compare nutritional composition with non-GM varieties, and examine the stability of genetic insertions across generations (Goodman, 2014).

Regulatory evaluation of GM amaranth requires careful consideration of the plant’s biology and its interactions within ecosystems. Beyond standard regulatory requirements, amaranth poses unique challenges due to the presence of closely related wild and weedy species (Anuradha et al., 2023) and its predominantly allogamous reproductive system (Anuradha et al., 2023; Bansal and Kaur, 2025; Sokolova et al., 2024). These characteristics necessitate comprehensive assessment of gene flow risks to wild populations (Anuradha et al., 2023; Bansal and Kaur, 2025), the potential for facilitating the evolution of competitive weedy species (superweeds), impacts on biodiversity, and the traceability and labelling of derived products (Anuradha et al., 2023). Ethical and commercial considerations also arise, including consumer acceptance, public perceptions of GMOs, and risks associated with technology concentration (Anuradha et al., 2023; Bansal and Kaur, 2025; Goodman, 2014). The absence of commercial GM amaranth cultivars is attributable not only to technical challenges in transformation but also to regulatory and market constraints (Anuradha et al., 2023).

### 7.6 Challenges for scaling up transformation protocols

Scaling up transformation protocols from the laboratory to large-scale breeding applications faces multiple challenges (Joshi et al., 2018). Among these challenges, the marked dependence on genotype and explant stands out (Bansal and Kaur, 2025; Joshi et al., 2020), leading to drastic variations in transformation and regeneration rates. Furthermore, reduced post-transformation regeneration rates, especially in grain amaranth, remain a major bottleneck in obtaining stable transgenic lines (Yaroshko et al., 2023). Interlaboratory reproducibility is limited by inherent differences in explant physiological state, media composition, and culture conditions, which hinder protocol standardization (Yaroshko et al., 2023). The frequent occurrence of chimeric plants in techniques not based on somatic embryogenesis requires additional cycles of selection and stabilization (Joshi et al., 2020). Furthermore, tissue culture processes are often lengthy and expensive (Joshi et al., 2020), limiting their application in breeding programs with limited resources, as is often the case with orphan crops (Kumar et al., 2021). Taken together, these technical challenges explain why genetic transformation in amaranth is primarily used for functional validation studies and specialized metabolite production, rather than for generating commercial cultivars (Anuradha et al., 2023; Joshi et al., 2020; Yaroshko et al., 2023).

### 7.7 Successful stories and main obstacles to genetic improvement

Amaranth varieties have been improved through conventional selection, hybridization, and mutagenesis, leading to farmer adoption and notable gains. For instance, ‘Plainsman,’ developed in the United States, demonstrates enhanced yield, early maturity, and lodging resistance. Indian cultivars such as GA-1, GA-2, Suvarna, and Annapurna, as well as Peruvian cultivars Oscar Blanco, Noel Vietmeyer, and Alan Garcia, exhibit higher yields, greater adaptability, and improved disease resistance. These successes, achieved through targeted breeding, highlight the crop’s promise for agricultural improvement. Similarly, breeding programs in Asia have contributed notable advances, such as the release of varieties with heat and cold tolerance (e.g., ‘Tainung No. 1’ and ‘Tainung No. 2’ in Taiwan) (Priyadarsini et al., 2025). In addition to these conventional approaches, experimental research has produced transgenic lines that express genes for resistance to pests and pathogens (Sokolova et al., 2024), plants with tolerance to salinity or drought through the overexpression of transcription factors such as AhDOF-AI or AhNF-YC (Priyadarsini et al., 2025), and transgenic roots obtained through Agrobacterium rhizogenes that produce secondary metabolites of industrial or nutraceutical interest (Sokolova et al., 2024).

Despite these advances, several persistent obstacles hinder amaranth improvement. High heterozygosity challenges stable line development, floral structure limits controlled crosses, and unfavorable traits like seed shattering and plant height reduce harvest efficiency.

Additionally, challenges such as poor tissue culture response, inefficient transformation methods, limited genomic resources, restricted field evaluation, insufficient synergy between traditional and genomic techniques, regulatory barriers, and market constraints further obstruct progress.

In summary, amaranth has significant potential to enhance resilient, nutritious agriculture. Realizing this potential requires coordinated solutions to overcome technical, regulatory, and commercial barriers.

## 8 Future prospects

**Figure 7:**
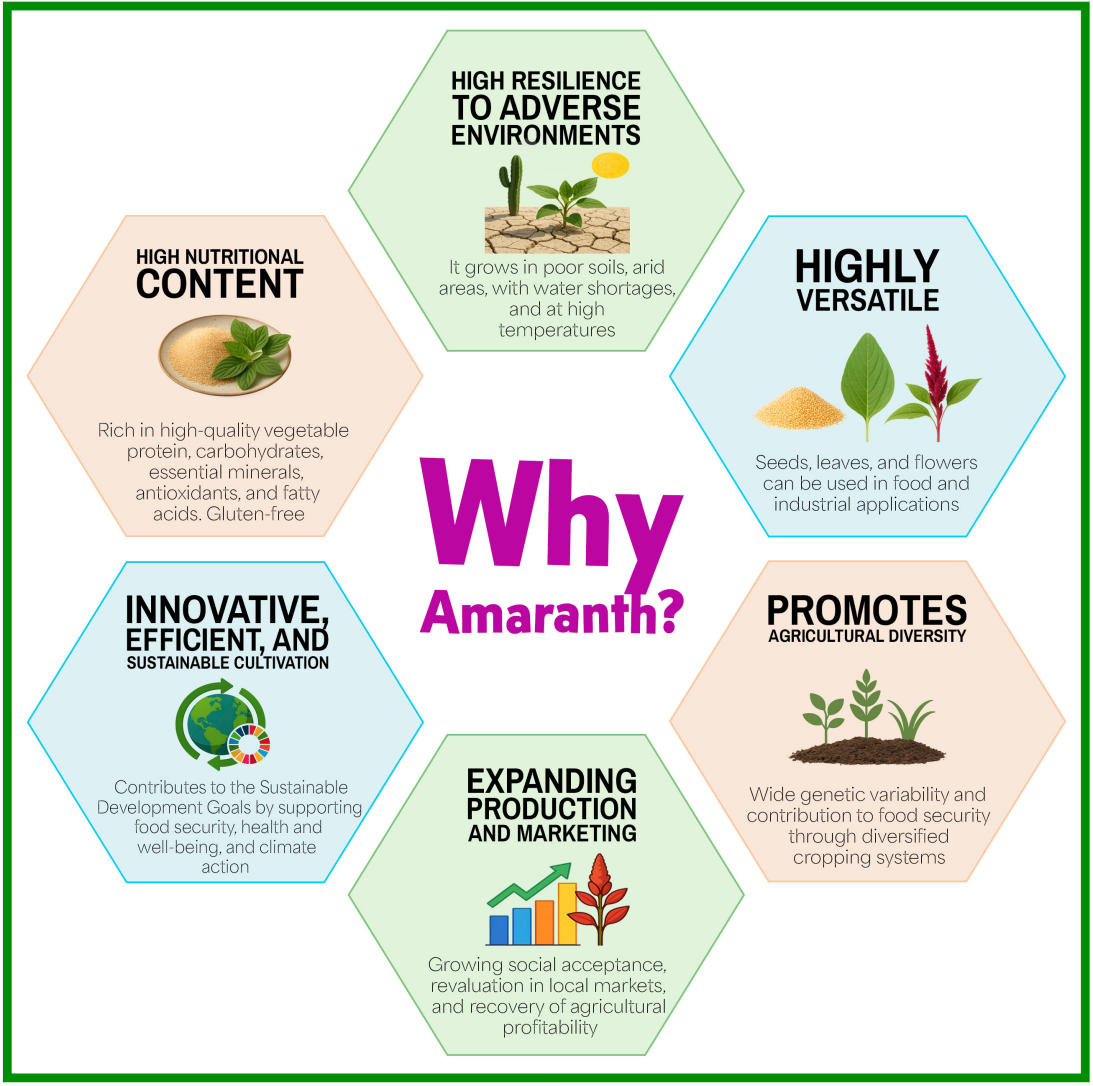
Key attributes for prospects on supporting the potential of *Amaranthus* spp. as a resilient and multifunctional crop.

Plants are fundamental to terrestrial ecosystems, serving as the primary producers in food webs and supplying essential energy. Of the estimated 374,000 plant species globally, approximately 80,000 have potential applications in food, feed, industry, or medicine; however, only 150–200 species account for the majority of human consumption (Christenhusz and Byng, 2016; Prescott-Allen and Prescott-Allen, 1990). Four staple crops—maize, potato, rice, and wheat—provide over 60% of global dietary energy (FAO, 2010). In contrast, many underutilized species, often referred to as orphan crops, receive limited research attention and funding despite their capacity to enhance nutrition, genetic diversity, and food security (Mukuwapasi et al., 2024) The FAO identifies amaranth as one such orphan crop highlighting its significant potential but also the absence of standardized agronomic protocols, phenotyping frameworks, genetic and molecular research, and improved varieties suitable for widespread cultivation (FAO, 2010). **Figure 7** illustrates the key attributes that justify the potential of *Amaranthus* spp. as an orphan crop with resilient and multifunctional features.

Realizing the potential of amaranth as an alternative crop requires prioritizing comprehensive research on its phenotypic, genetic, and molecular characteristics, as well as its tolerance to environmental stresses (Yadav and Yadav, 2024). Key research areas include assessing growth responses across diverse environmental conditions, analyzing nutrient and flour properties, and developing breeding strategies to improve yield stability, nutritional quality, and climate resilience. There is an increasing imperative for researchers, policymakers, and institutions to engage in interdisciplinary collaborations that advance amaranth research, ensuring the development of robust varieties and knowledge systems to address future climatic and nutritional demands (Kaur et al., 2024; Yadav and Yadav, 2024; Stetter et al., 2025).

The successful promotion and scaling of amaranth cultivation necessitate coordinated efforts across policy, research, and market sectors, underpinned by open data systems and participatory governance. Achieving effective scaling requires integrated pilot investments that connect breeding, localized agronomy, institutional procurement, and early-stage processing to generate evidence and inform national strategies (Mukuwapasi et al., 2024; Müller and Schneider, 2017). Strategic policy interventions can position amaranth within climate-resilient, underutilized crop portfolios in national food system strategies, Nationally Determined Contributions (NDCs), and adaptation plans (Yadav and Yadav, 2024). The expansion of trade and market development depends on regulations for seed registration, varietal release, food safety standards, and phytosanitary requirements. Incentives for land and water use should support drought-tolerant, low-input, and intercropping systems that favor amaranth (Yadav and Yadav, 2024). Additionally, policies must protect traditional knowledge and seed sovereignty, ensuring equitable benefit-sharing with indigenous and local communities engaged in breeding, cultivation, and commercialization (Kloppenburg, 2010; FAO, 2019; Anuradha et al., 2023; Rastogi and Shukla, 2013).

Conserving and characterizing Amaranthus genetic diversity is fundamental for pre-breeding initiatives that target improvements in drought and heat tolerance, lodging resistance, and the reduction of anti-nutritional factors (Anuradha et al., 2023; Stetter and Schmid, 2020). Breeding programs should prioritize the development of dual-purpose cultivars capable of delivering both competitive grain yields and substantial leaf biomass (Dinssa et al., 2018). Conducting multi-location trials is essential to optimize agronomic management in *Amaranthus* (Stetter and Schmid, 2020). In addition, standardized nutritional and processing datasets, together with validated protocols for puffing, extrusion, milling, and leaf blanching, should be made available through open platforms and decision-support tools to accelerate breeding and utilization (Rastogi and Shukla, 2013; Anuradha et al., 2023).

Successful adoption will require context-specific packages that distinguish grain vs leafy types, rainfed vs irrigated systems, and smallholder vs mechanized farms (Kaur et al., 2024; Mukuwapasi et al., 2024). Strengthened seed systems—through community seed banks, quality-declared seed and public–private multiplication—combined with blended extension approaches (farmer field schools, peer networks, digital advisory) will increase access to adapted varieties and best practices (Altaye et al., 2024). Appropriate small-scale mechanization (threshers, dryers), low-cost storage and improved cold-chain and hygiene for fresh leaves will reduce losses and add value across value chains (Mng’omba, 2025).

Developing a diversified product portfolio will increase demand and help stabilize the market. Culturally relevant products such as flatbreads, porridges, snacks, blended flours, baby foods, and leafy vegetables provide multiple market entry points (Kahlon et al., 2014). Implementing quality standards and grading for grain purity, moisture content, and leaf freshness, alongside certifications such as organic, fair trade, and gluten-free, can facilitate market differentiation (Amaranth Market Size Share Industry Analysis Report, 2023). Establishing aggregation hubs, forward contracts, and stronger connections among farmer organizations, processors, and institutional buyers will help stabilize supply and reduce market volatility (Mukuwapasi et al., 2024; Mng’omba, 2025; FAO, 2019). Nutrition campaigns co-developed with community leaders and chefs that highlight protein quality, micronutrient density, and gluten-free properties, together with price-stabilization mechanisms, can increase consumer demand and protect smallholders from seasonal price fluctuations (Anuradha et al., 2023; Kaur et al., 2024; Managa and Nemadodzi, 2023).

Nevertheless, rapid scaling introduces ecological, economic, and socio-cultural risks. Large-scale monocropping can reduce biodiversity, increase pest and disease pressures, and deplete soil nutrients, particularly where intensive leaf harvesting occurs without crop rotations (Kaur et al., 2024; Yadav and Yadav, 2024). Certain *Amaranthus* species possess invasive potential, necessitating effective weed management strategies (Emmanuel and Babalola, 2022). From an economic perspective, expansion without coordinated market development may result in oversupply, price volatility, and the marginalization of smallholders as value chains consolidate (Okonkwo-Emegha et al., 2025). Export-driven demand could divert grain from local consumption, while the loss of landraces, diminished access to leafy greens in traditional diets, and changing gender roles represent potential socio-cultural impacts (FAO, 2021). Issues of seed sovereignty, intellectual property, and benefit-sharing require robust governance to prevent inequities (European Commission, 2009; Müller and Schneider, 2017).

International collaborations reflect increasing engagement in amaranth research and development. The European Commission FP6 project AMARANTH: FUTURE-FOOD established foundational research networks and facilitated commercialization efforts (Kaur et al., 2024). The FAO’s One Country, One Priority Product (OCOP) program elevated amaranth to national priority status in Mexico, linking agroecological transition with food sovereignty objectives (FAO, 2021). In Africa, systems modelling of value chains (Dizyee, 2020), breeding and promotion partnerships (KALRO with Ripple Effect), and Kenya’s inclusion of amaranth in the Power of Diversity Funding Facility (Murigi, 2025) exemplify coordinated policy and research initiatives. In Tanzania, WorldVeg-developed varieties have achieved widespread adoption among 67% of producers (Food and Agriculture Organization of the United Nations, 2021), with yields up to 6.1 t·ha^-1^ compared with local varieties (Wanyama et al., 2023). In China, research is expanding into nutraceutical, pharmaceutical, and cosmetic applications (Aditya et al, 2020).

It can be said that, these regionally distinct yet complementary initiatives collectively form the foundation for a future roadmap to integrate amaranth into climate-resilient food systems. In Africa, efforts center on value chain development, neglected crop policy, and resilience through systems modeling, ACP–EU briefs, and symposia, resulting in income-diversification pilots and policy briefs (ILRI 2015; BecA ILRI 2016; WorldVeg 2023; ECHO East Africa 2018; Westlake, 2014). Latin America prioritizes national recognition and biocultural value, with OCOP designation and regional knowledge exchange generating strategic outlines (FAO OCOP Mexico 2021; FAO Webinar 2022). Asia emphasizes agronomy and functional food systems, with reviews and adaptation notes producing climate-smart guidance and dual-purpose crop strategies (ECHO Asia 2019; Naveen et al., 2024; Kioumarsi et al., 2025). Europe, building on FP6, provides market analyses and exploitation tools to support consumer demand expansion (European Commission, 2009; Müller and Schneider, 2017). North America focuses on global networking and Indigenous perspectives through conferences, outreach, and international collaboration (Amaranth Institute 2022; Rutgers University 2021; Tennessee State University 2022; World Renew 1998; WeEffect 2020).

In summary, amaranth has the potential to transition from an underutilized species to a significant contributor to climate adaptation, food security, and sustainable rural development. Achieving scale will require coordinated policies, robust research, context-specific farmer adoption, and competitive markets, while also protecting traditional knowledge, seed sovereignty, and equitable benefit-sharing. Through open data, participatory governance, and targeted investment, Amaranth offers a strategic opportunity to strengthen agricultural resilience, empower smallholders, and diversify nutrition in response to accelerating climatic and socio-economic changes.
